# Alleviating Pb Toxicity in Rice–Soil Systems by GSNO-Encapsulated Chitosan–Biochar/S-nZVI Composites: Sustained Release of NO and the Stabilization of Pb

**DOI:** 10.3390/molecules31162761

**Published:** 2026-08-08

**Authors:** Fanjiang Yang, Chunfang Tang, Yutong Zhang, Xinyu Xu, Meifang Li, Chengyun Zhou, Lei Qin, Jiaqin Deng, Xinjiang Hu, Xiaoxi Cai, Shaoheng Liu

**Affiliations:** 1 School of Ecology and Environment, Central South University of Forestry and Technology, Changsha 410004, China; 2 College of Environmental Science and Engineering, Hunan University, Changsha 410082, China; 3 School of Landscape Architecture, Central South University of Forestry and Technology, Changsha 410004, China; 4 College of Chemistry and Material Engineering, Hunan University of Arts and Science, Changde 415000, China

**Keywords:** rice, Pb stress, CS-based composites, GSNO encapsulation, NO sustained-release

## Abstract

Lead (Pb) accumulation in paddy soils promotes its transfer to rice grains, posing persistent risks to food safety and human health. Here, we developed a GSNO-encapsulated chitosan–biochar-supported sulfidized nanoscale zero-valent iron composite (GSNO-CS@S-nZVI/BC) to integrate sustained nitric oxide (NO) delivery with Pb immobilization in rice–soil systems. The novelty of this strategy lies in coupling a biocompatible NO donor with a Pb-reactive CS@S-nZVI/BC matrix to simultaneously alleviate Pb-induced physiological stress in rice and suppress Pb migration from soil to edible grains. In hydroponic experiments, Pb exposure reduced rice fresh weight, dry weight, and chlorophyll levels by 71.2%, 55.6%, and 75.1%, respectively. Compared with Pb treatment alone, GSNO-CS@S-nZVI/BC increased rice dry weight and chlorophyll levels by 68.5% and 260%, respectively, while decreasing Pb concentrations in roots and shoots by 78.2% and 85.8%. In soil pot experiments, GSNO-CS@S-nZVI/BC reduced Pb concentrations in roots, shoots, panicles, and grains by 57.6%, 57.0%, 40.3%, and 64.3%, respectively. It also decreased the grain Pb bioconcentration factor by 80.7%, reduced the soil-to-root Pb translocation factor by 84.41%, and lowered the grain Pb bioconcentration factor to 0.043 ± 0.007. Mechanistically, GSNO-CS@S-nZVI/BC promoted root iron plaque formation by 176.1% and increased Pb adsorption by root iron plaque by 156.9%, while decreasing the oxidizable Pb fraction in soil by 11% and increasing the reducible Pb fraction by 10%. These results indicate that GSNO-CS@S-nZVI/BC mitigates Pb toxicity through sustained NO release, enhanced root iron plaque-mediated Pb sequestration, and soil Pb stabilization. Overall, this nano-enabled amendment provides a promising strategy for reducing Pb transfer from contaminated paddy soils to rice grains and improving the safe utilization of Pb-contaminated farmland.

## 1. Introduction

Human activities such as mining and smelting, solid waste disposal, and sewage irrigation have led to a serious problem of lead (Pb) contamination in the global soil environment [1]. Pb, as a highly toxic heavy metal, is of particular concern in soil due to its persistence, bioaccumulation potential, and irreversible impact on soil quality [2,3]. As a global staple crop, rice is increasingly threatened by the accumulation of heavy metals in agricultural soils. The deleterious effects of Pb on rice mainly include reducing seed germination, inhibiting the growth of roots and shoots, and resulting in a decrease in biomass. Additionally, Pb stress can disrupt the mineral nutrient balance, leading to a decrease in cell division and photosynthesis in rice [4]. Soil Pb pollution has been demonstrated to have harmful effects on the quality and yield of rice. Pb has been shown to pose a threat to human health through the process of bioconcentration [5], which can result in a diminution of the immune system and a consequent array of symptoms, such as nervous system dysfunction, dizziness, insomnia, vomiting, and abdominal distension [6]. In addition, the International Agency for Research on Cancer (IARC) of the World Health Organization has classified Pb and its compounds as Group 2B carcinogens [7]. Therefore, seeking a green and environmentally friendly technology to effectively alleviate the adverse effects of Pb stress on rice is of great significance for food safety and human health.

Exogenous nitric oxide (NO) is effective in alleviating abiotic stresses such as salt [8], drought [9], cold [10], and heavy metals [11] in a variety of plant species. In addition, the interaction between NO and inorganic nitrogen sources in the soil can promote plant growth and enhance plant tolerance [12]. However, the direct application of NO to plants is difficult due to the gaseous nature of NO. Additionally, NO has a short half-life (1–5 s) in the body and is easily inactivated by reacting with oxygen or hemoglobin, but it can be continuously replenished to the plant cells by exogenous NO donors to alleviate the adverse effects of heavy metal stress on rice [13].

NO donors are compounds that generate NO without requiring the catalysis by NO synthase in living organisms, via either spontaneous decomposition or interaction with other substances. Currently, most studies select sodium nitroprusside (SNP) as the typical exogenous NO donor to alleviate heavy metal contamination [14]. However, SNP is easily decomposed by hemoglobin into cyanide, and cyanide is partially detoxified by the liver and kidneys, turning into thiocyanate [15]. Therefore, it was necessary to find a safe and reliable NO donor. S-nitrosoglutathione (GSNO), produced through the nitrosation of reduced glutathione (GSH), serves not only as an effective NO donor but also exhibits non-toxic and environmentally friendly properties, which spontaneously releases NO through cleavage of the S-N bond [9,16]. Nevertheless, it is noteworthy that GSNO, similar to SNP, exhibits inherent thermal and photochemical instability [9]. Short-term high-concentration release can also trigger the autotoxicity of NO, thereby hindering its application in agriculture [9].

Nanomaterials can effectively prevent the premature decomposition of NO donors in the physiological environment by encapsulating them and forming a physical barrier, which can avoid non-enzymatic catalytic decomposition and the rapid release of NO, thereby enhancing the stability of the NO donors [17]. Ma et al. reported that the chitosan (CS)-based nanoparticles encapsulating GSNO could effectively prolong the release time of NO [18]. Methela et al. also achieved the purpose of extending the release time by encapsulating GSNO within CS-based nanoparticles, in order to prevent the rapid decomposition of NO [19]. Therefore, CS-based nanocomposites are commonly used as a carrier for encapsulating NO donors [20], which is a type of low-cost and environmentally friendly material with characteristics such as biodegradability, biocompatibility, and large surface area [21]. Furthermore, the abundant amino (-NH_2_) and hydroxyl (-OH) groups on the CS molecular chains can easily chelate with various heavy metal ions [22,23]. However, pure CS exhibits limited adsorption capacity and poor selectivity for heavy metal ions [24]. Additionally, the encapsulation of NO donors may block the adsorption sites on CS, further reducing its metal-binding efficiency. Zero-valent nano-iron (nZVI) not only can stimulate the formation of iron plaque on the root surface but has also been proven to be widely used for removing heavy metals from aqueous solutions [25]. Research has shown that CS as a stabilizer could effectively alter the surface charge of nZVI, increasing electrostatic repulsion and reducing aggregation and sedimentation [26], thereby synergistically enhancing the pollutant removal efficiency. In addition, biochar (BC) has the advantages of low cost, porous structure, high specific surface area, and high adsorption capacity [27], which is regarded as one of the best carriers for the dispersion of nZVI and has great potential for the immobilization of heavy metals [28].

Carbonaceous materials are widely recognized for their critical roles in environmental remediation, primarily due to their high surface area, tunable porosity, and versatile surface chemistry. Recent studies have demonstrated that advanced carbon architectures, such as ordered mesoporous carbons prepared from different natural precursors, nitrogen-doped bamboo-like carbon nanotubes with encapsulated metal particles, and hollow-carbon or carbon-encapsulated metal structures derived from metal–organic frameworks, can achieve excellent adsorption and catalytic performance [29,30,31]. However, these materials often rely on multi-step synthesis involving specialized precursors, high-temperature treatment, or template methods. In the context of large-scale soil amendment, biochar produced from abundant biomass via simple pyrolysis offers a more practical alternative. It retains sufficient surface area and functional groups while serving as an effective support for the dispersion of nZVI and as a matrix for GSNO encapsulation, thereby enabling the dual functions of Pb immobilization and sustained NO release required in rice–soil systems. Li et al. also found that BC-supported nZVI materials had excellent removal effects on Pb in water. The main removal mechanisms involve reduction, complexation, and co-precipitation [32].

Current research on GSNO-mediated sustained NO release has primarily focused on biomedical applications, whereas its potential for mitigating heavy metal stress in crop-soil systems remains insufficiently explored [33]. Existing plant-related studies have mainly used GSNO alone or CS-based nanoparticles as NO delivery systems, with limited consideration of whether the carrier itself can simultaneously immobilize heavy metals [18,34]. Moreover, there are relatively few reports on the use of excellent GSNO carriers in the field of treating heavy metal pollution in rice. Therefore, it is necessary to conduct research on developing a high-quality GSNO carrier and exploring its synergistic effect with GSNO to alleviate Pb stress in rice from multiple aspects.

In this study, we developed a GSNO-encapsulated chitosan–biochar-supported sulfidized nanoscale zero-valent iron composite (GSNO-CS@S-nZVI/BC) through ionotropic gelation [35]. The technical novelty of this system is that CS@S-nZVI/BC serves not only as a matrix for GSNO encapsulation and sustained NO delivery, but also as an active Pb-immobilizing amendment through the combined effects of chitosan, biochar, and S-nZVI. Based on these properties, we hypothesized that GSNO-CS@S-nZVI/BC would alleviate Pb toxicity in rice–soil systems through two complementary pathways: (i) sustained NO release would improve rice physiological tolerance to Pb stress and promote root iron plaque formation, and (ii) the CS@S-nZVI/BC matrix would stabilize Pb in the growth medium and reduce its bioavailability, thereby limiting Pb uptake and translocation from roots to aboveground tissues and grains.

To test this hypothesis, hydroponic and soil pot experiments were conducted to evaluate the effects of GSNO-CS@S-nZVI/BC on Pb-stressed rice. The measurable targets included rice growth parameters, biomass, chlorophyll levels, Pb concentrations in roots, shoots, panicles, and grains, Pb bioconcentration and translocation factors, Pb fractions in soil, and the formation and Pb adsorption capacity of root iron plaque. By integrating physiological indicators, Pb uptake and transport parameters, soil Pb speciation, and root iron plaque characteristics, this study aimed to clarify whether GSNO-CS@S-nZVI/BC can function as a dual-action amendment for both Pb stress alleviation and Pb migration control in rice–soil systems. The findings are expected to provide a mechanistic and application-oriented basis for the safe utilization of Pb-contaminated paddy soils.

## 2. Results and Discussions

### 2.1. Characterization of the Materials

**Figure 1:**
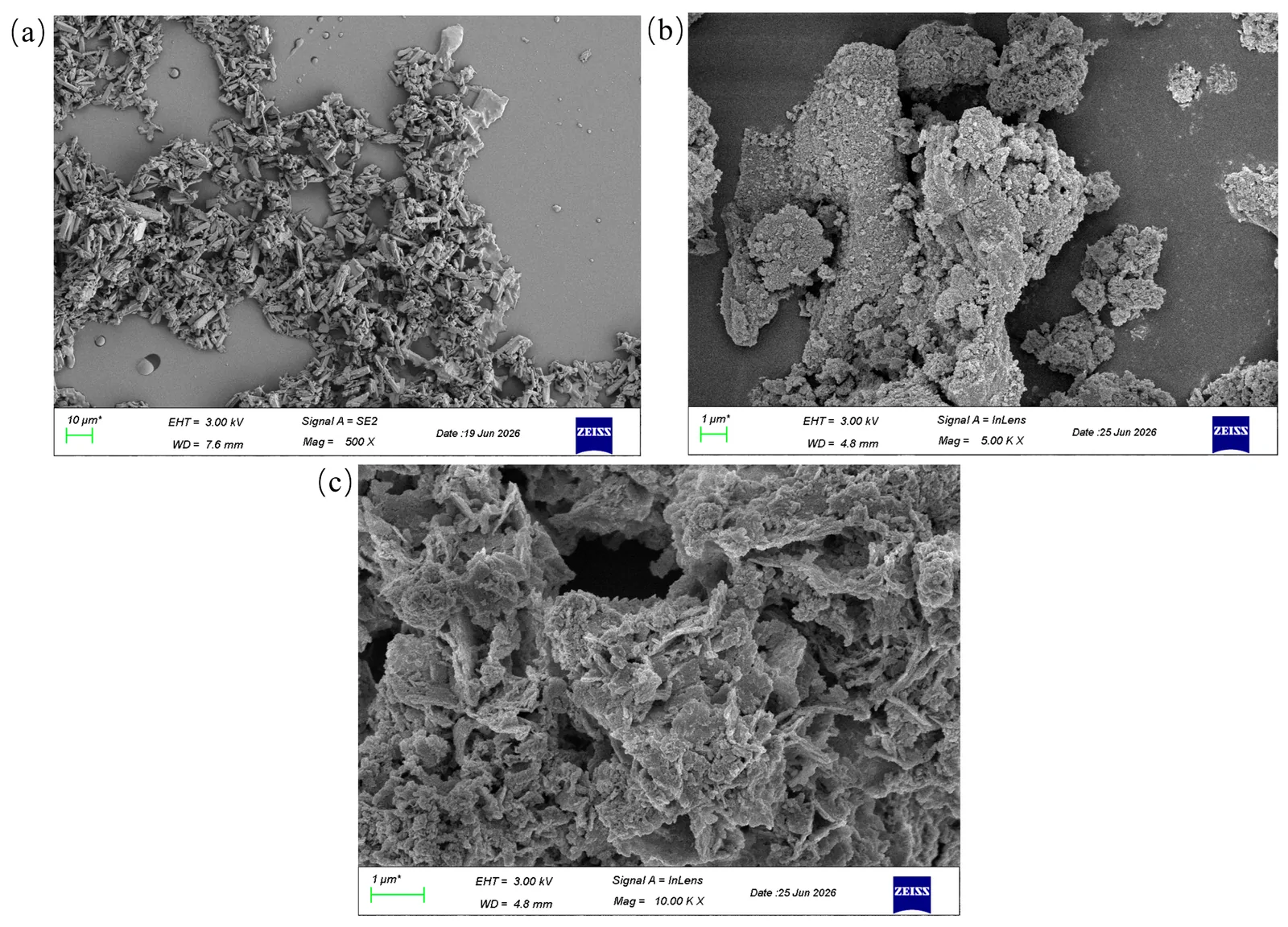
SEM images showing the morphological characteristics of (**a**) pure GSNO, (**b**) CS@S-nZVI/BC carrier, and (**c**) GSNO-CS@S-nZVI/BC composite at different magnifications. The asterisk (*) after the scale bar unit is a standard annotation automatically generated by the ZEISS SmartSEM software and has no special meaning.

Scanning electron microscopy was performed to visualize the structural changes associated with the encapsulation of GSNO into the CS@S-nZVI/BC matrix. As shown in Figure 1a, pure GSNO displayed a typical rod-like and flaky crystalline morphology with irregular aggregation. This appearance is consistent with the precipitation synthesis of GSNO from GSH and sodium nitrite (NaNO_2_) under acidic conditions, as commonly reported in studies involving GSNO as an NO donor.

The CS@S-nZVI/BC carrier (Figure 1b) exhibited an irregular, highly aggregated structure with a rough and porous surface. This morphology reflects the biochar framework providing high surface area and porosity, together with S-nZVI nanoparticles distributed across the surface, which is in good agreement with previously characterized biochar-supported nZVI composites [35].

In the GSNO-CS@S-nZVI/BC (Figure 1c), the overall aggregated framework was retained; however, the surface exhibited distinctly modified features, including layered and folded sheet-like structures with significantly increased roughness at higher magnification. These changes indicate successful encapsulation of GSNO within the chitosan matrix via ionotropic gelation. The formation of an additional organic coating layer on the carrier surface is evident, leading to altered surface texture and enhanced particle aggregation compared to the bare CS@S-nZVI/BC. Similar morphological transitions from porous or particulate carriers to coated or layered composites have been widely observed in the literature on chitosan-encapsulated NO donors and bioactive molecules loaded onto carbon-based or inorganic supports, where such surface modification is directly linked to controlled release behavior and improved stability.

The retention of the porous biochar and S-nZVI features from the carrier, combined with the emergence of coated and layered surface characteristics in the final material, provides clear visual confirmation of successful GSNO integration. This structural evolution is expected to contribute to the sustained NO release profile while preserving the Pb immobilization capacity of the S-nZVI/BC component, as demonstrated in subsequent functional evaluations.

**Figure 2:**
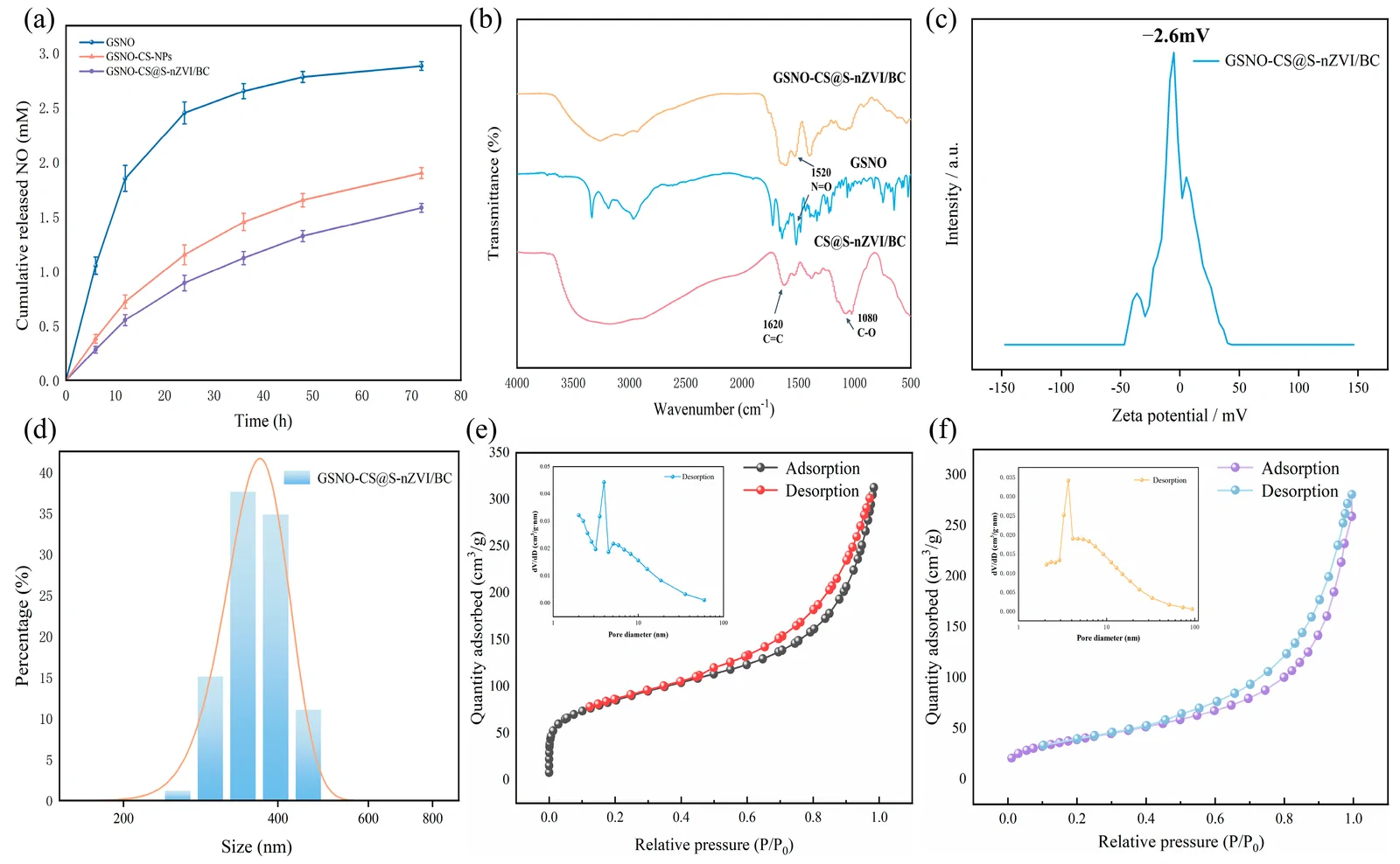
Physicochemical characterization and in vitro NO release kinetics of the synthesized materials. (**a**) Time-dependent NO release profiles of free GSNO, GSNO-CS, and GSNO-CS@S-nZVI/BC at a dosage of 1 g L^−1^. (**b**) Fourier transform infrared spectra of the individual components and the final composite. (**c**) Zeta potential and (**d**) particle size distribution of the GSNO-CS@S-nZVI/BC. Nitrogen adsorption–desorption isotherms and corresponding pore size distribution curves of (**e**) CS@S-nZVI/BC and (**f**) GSNO-CS@S-nZVI/BC.

Following the morphological observation by SEM, the chemical structures and the successful loading of the NO donor were further verified by FTIR spectroscopy (Figure 2b). Compared with the spectra of the pristine components, the GSNO-CS@S-nZVI/BC composite exhibited characteristic absorption bands corresponding to the N=O and S–N stretching vibrations of GSNO [36], alongside the typical amide and hydroxyl peaks of the CS matrix [37]. The slight shifts in wavenumbers and variations in peak intensities indicate strong intermolecular interactions, such as hydrogen bonding and electrostatic forces, confirming the successful encapsulation of GSNO within the CS-based network. Furthermore, quantitative analysis revealed a high encapsulation efficiency of 87.92%. This highly effective encapsulation not only protects the environmentally sensitive GSNO from premature photolytic and thermal degradation but also lays a solid foundation for its sustained release in the soil–rice system.

The physicochemical properties of the composites in aqueous environments, which largely dictate their environmental behavior and bioavailability, were evaluated via hydrodynamic particle size and zeta potential measurements (Figure 2c,d). The GSNO-CS@S-nZVI/BC displayed a favorable sub-micron hydrodynamic diameter and a specific surface charge profile. The appropriate particle size ensures a sufficient specific surface area, providing abundant reactive sites for Pb^2+^ adsorption and facilitating the subsequent formation of root iron plaque. Meanwhile, the zeta potential reflects the surface charge characteristics that govern electrostatic interactions with heavy metal ions. The specific surface charge of the composite promotes a strong affinity and complexation with positively charged Pb^2+^, thereby enhancing Pb immobilization in the rhizosphere. More importantly, these optimized physicochemical properties endow the composite with excellent biocompatibility and rhizosphere retention.

N_2_ adsorption–desorption isotherms (Figure 2e,f and Table 1) revealed that CS@S-nZVI/BC possessed a high BET surface area of 299.22 m^2^/g and a total pore volume of 0.484 cm^3^/g. After GSNO encapsulation, the BET surface area of GSNO-CS@S-nZVI/BC decreased to 143.31 m^2^/g, and the total pore volume declined to 0.383 cm^3^/g, while the average pore size increased from 6.47 nm to 10.69 nm. This moderate decrease in surface area and pore volume was consistent with the SEM observation that GSNO was successfully encapsulated within the chitosan matrix, which partially occupied the pores. Nevertheless, the composite retained a well-developed mesoporous structure. Concurrently, the particle size distribution (Figure 2d) showed that GSNO-CS@S-nZVI/BC was dominated by sub-micron particles, in which the irregular porous morphology of CS@S-nZVI/BC evolved into a more aggregated structure featuring layered and folded surface characteristics after GSNO incorporation. Comparison of the particle size distribution curves of CS@S-nZVI/BC (Figure S1) and GSNO-CS@S-nZVI/BC (Figure 2d) showed that GSNO modification led to an overall decrease in particle size. This reduction is attributed to the abundant carboxyl and amino groups of GSNO, which significantly alter the surface charge of the particles and thereby diminish the size of aggregates in aqueous suspension. The resulting smaller dispersed size implies higher reactivity and enhanced penetration capability, further confirming the successful loading of GSNO.

**Figure 3:**
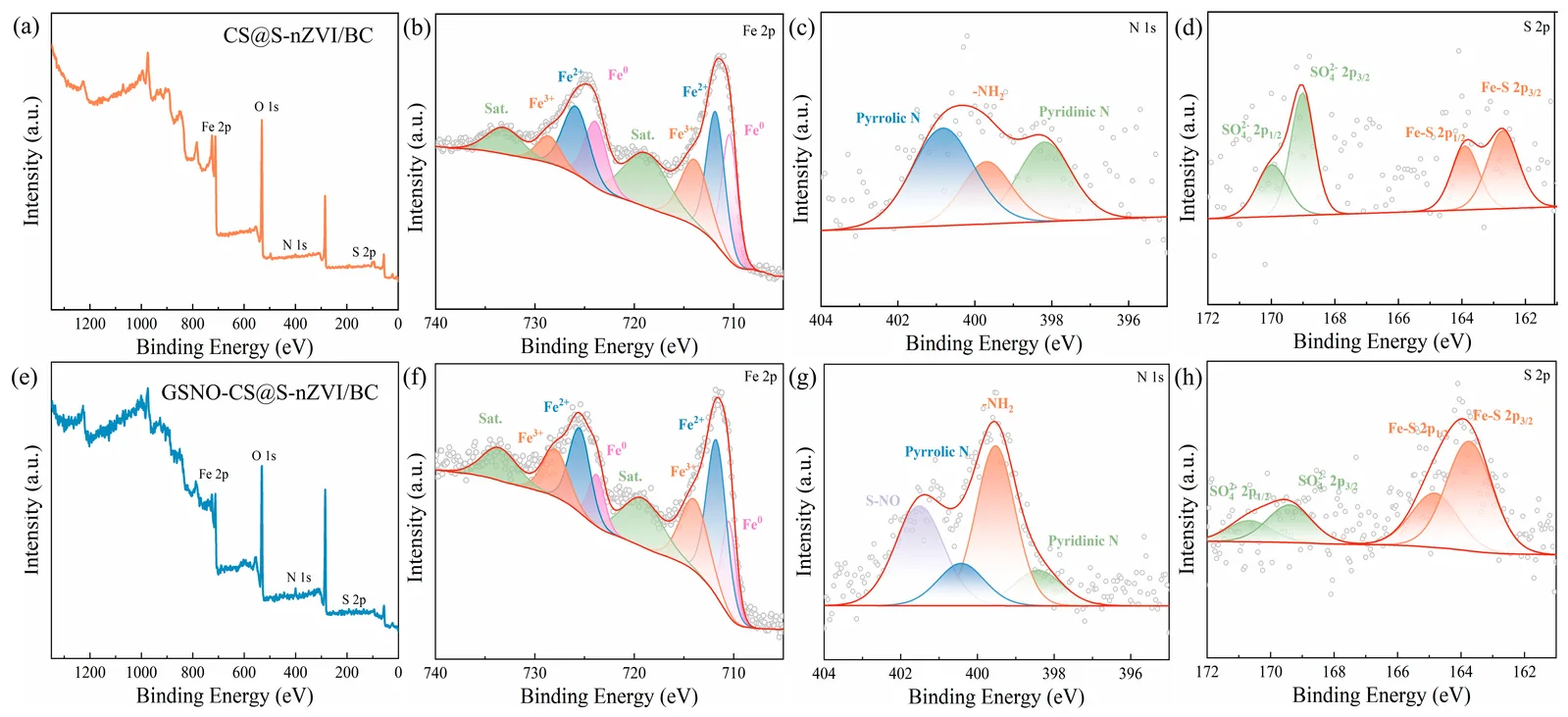
XPS spectra of CS@S-nZVI/BC and GSNO-CS@S-nZVI/BC. (**a**,**e**) Survey spectra; (**b**,**f**) high-resolution Fe 2p spectra; (**c**,**g**) high-resolution N 1s spectra; (**d**,**h**) high-resolution S 2p spectra of CS@S-nZVI/BC (**a**–**d**) and GSNO-CS@S-nZVI/BC (**e**–**h**), respectively.

XPS analyses were conducted to further confirm the successful retention of zero-valent iron, the occurrence of sulfidation, and the effective encapsulation of GSNO. High-resolution Fe 2p spectra of both CS@S-nZVI/BC and GSNO-CS@S-nZVI/BC clearly displayed characteristic peaks of Fe^0^, Fe^2+^, and Fe^3+^ (Figure 3b,f), demonstrating that zero-valent iron was successfully formed and well preserved after sulfidation and GSNO loading. The coexistence of Fe^2+^/Fe^3+^ indicates the formation of a typical core–shell structure, which is beneficial for balancing reactivity and stability. Meanwhile, the S 2p spectra (Figure 3d,h) showed distinct Fe–S signals accompanied by sulfate peaks, providing direct evidence for successful sulfidation.

In the N 1s spectrum of GSNO-CS@S-nZVI/BC (Figure 3g), the appearance of an S–NO component compared with CS@S-nZVI/BC further verified the successful incorporation of GSNO. This spectroscopic evidence was highly consistent with the FTIR results, which retained the characteristic N=O and S–N stretching vibrations of GSNO.

Collectively, the XPS, BET, SEM, and FTIR results mutually reinforced the successful construction of GSNO-CS@S-nZVI/BC, thereby laying a solid foundation for its superior performance in rice–soil systems.

### 2.2. In Vitro Nitric Oxide Release from Free and Matrix-Encapsulated GSNO

The in vitro NO release kinetics of free GSNO, GSNO-CS-NPs, and GSNO-CS@S-nZVI/BC were evaluated at the application concentration of 1 g L^−1^ under conditions representative of the hydroponic experiments. Cumulative NO release was monitored over 72 h by UV–Vis spectrophotometry at 336 nm. As shown in Figure 2a, free GSNO exhibited a rapid initial burst, releasing 1.05 ± 0.08 mM within 6 h and reaching 2.45 ± 0.10 mM by 24 h, with cumulative release approaching 2.88 ± 0.04 mM at 72 h. Encapsulation in chitosan-based matrices substantially attenuated the initial burst and enabled sustained release. GSNO-CS-NPs released only 0.38 ± 0.04 mM in the first 6 h, attaining 1.15 ± 0.09 mM at 24 h and 1.90 ± 0.05 mM at 72 h. The GSNO-CS@S-nZVI/BC composite showed the slowest release profile, with 0.28 ± 0.03 mM released within 6 h, 0.89 ± 0.07 mM at 24 h, and 1.58 ± 0.04 mM at 72 h. Significant differences in cumulative NO release were observed among the three formulations at all time points after 6 h (*p* < 0.05).

The rapid decomposition of free GSNO is consistent with its inherent thermal and photochemical instability. Encapsulation within the chitosan matrix effectively reduced the initial burst and prolonged NO delivery over 72 h. These findings align with previous reports that chitosan encapsulation of GSNO significantly slows release kinetics and minimizes autotoxicity risks associated with high local NO concentrations [36]. Notably, GSNO-CS@S-nZVI/BC provided more sustained release than GSNO-CS-NPs, indicating that incorporation of the S-nZVI/BC component further modulates the release profile. This sustained NO delivery is expected to support prolonged alleviation of Pb stress in rice while reducing the potential for NO overaccumulation.

Although the in vitro release assay was performed under dark conditions to establish a reproducible baseline, GSNO was known to be particularly susceptible to light-induced decomposition in open environments. Previous studies have shown that free GSNO undergoes rapid photochemical degradation, whereas encapsulation within a chitosan matrix creates a physical barrier that markedly reduces light access to the S–N bond and thereby enhances photostability [9,36]. In the current composite materials, the additional biochar carriers further enhance the attenuation effect of light.

Moreover, under the soil-application regime used in this study (root irrigation of the composite suspended in nutrient solution, followed by incorporation into the flooded soil), the majority of particles are distributed within the root zone and soil matrix, where soil particles and the water layer themselves act as effective light shields. The clear alleviation of Pb toxicity together with the marked reduction in Pb accumulation in grains observed in the soil pot experiments under natural daylight further support that the CS@S-nZVI/BC matrix can maintain functional photoprotection under realistic paddy conditions.

Nevertheless, we acknowledge that the current evidence remains somewhat limited, as prolonged surface exposure under intense field sunlight could still accelerate NO release relative to the dark laboratory profile. For future field-scale applications, root-zone incorporation via nutrient-solution irrigation combined with late-afternoon or evening application and retention of the split-dose strategy already employed in this study is recommended to maximize photoprotection, while dedicated photostability tests under controlled light intensities and continuously flooded conditions would help refine application timing for large-scale use.

### 2.3. NO Sustained Release for Pb Stress Alleviation in Hydroponic Experiment

#### 2.3.1. Growth Characteristics of Rice in Hydroponic Experiment

**Figure 4:**
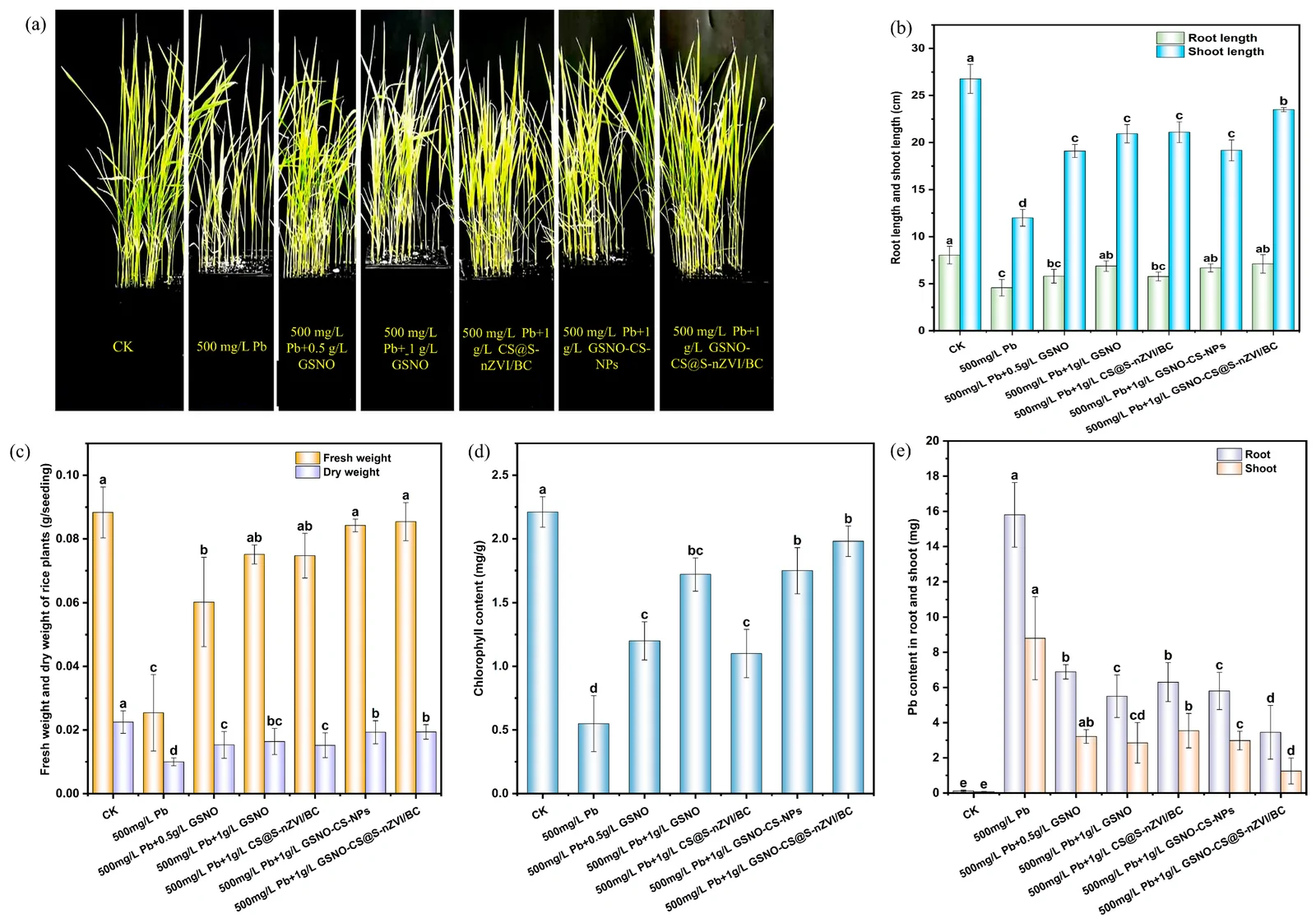
Growth characteristics of rice seedlings under hydroponic conditions (**a**); shoot and root lengths (**b**), fresh and dry weights (**c**), chlorophyll levels (**d**), and Pb content in root and shoot (**e**) of rice in hydroponic experiment. Different lowercase letters above the bars indicate statistically significant differences (*p* < 0.05). Bars sharing the same letter are not significantly different.

The rice growth morphology is shown in Figure 4a. Compared with the control group seedlings (CK group), a single treatment with 500 mg/L Pb significantly inhibited the growth of rice. However, as the concentration of GSNO increased, the inhibitory effect on rice growth gradually lessened. The addition of CS@S-nZVI/BC composite materials could also markedly mitigate the adverse influences of Pb stress on the growth of rice shoot than that of a single Pb treatment. Based on our previous research, CS@S-nZVI/BC showed good adsorption and immobilization effects on Pb in aqueous solutions. Therefore, CS@S-nZVI/BC could reduce the impact of Pb on the growth of rice seedlings [35]. It was worth noting that, after the GSNO was encapsulated by CS or CS@S-nZVI/BC materials, the growth condition of rice was significantly better than that of the treatment using GSNO alone. It indicated that the sustained release of NO was more effective in alleviating Pb stress on rice seedling growth than its free release. However, by comparing the two NO sustained-release materials, it was easy to find that the rice development treated with GSNO-CS@S-nZVI/BC was better than that treated with GSNO-CS-NPs. The finding demonstrated that encapsulating GSNO with the CS@S-nZVI/BC composite exhibited superior efficacy in mitigating Pb stress in rice compared to CS-only encapsulation systems. This might be due to the fact that, compared with CS nanomaterials, the CS@S-nZVI/BC composite material contained multiple components that could remove heavy metals or alleviate heavy metal stress for plants [38,39,40], thus playing a protective role for rice in the presence of Pb pollution.

The root length and shoot length of rice are shown in Figure 4b. Compared to the CK treatment, the inhibition rates of a single Pb treatment were 43.1% on root length and 55.2% on shoot length (*p* < 0.05). In contrast to the single Pb treatment, the shoot length of the GSNO treatment group was slightly longer and increased with the increase in GSNO application amount. Research has found that GSNO may inhibit heavy metal uptake by forming complexes with them, and GSNO can be taken up by the root and transported to the upper parts through the phloem [41]. This also indicated that exogenous NO alleviated the stress effect of Pb to a certain extent, and the effects of different concentrations of exogenous NO on alleviating Pb stress in rice were different. In addition, CS@S-nZVI/BC treatment significantly alleviated the inhibitory effect of Pb stress on rice stem growth (*p* < 0.05). Compared with using GSNO alone, embedding GSNO in the CS@S-nZVI/BC composite material was more conducive to alleviating the inhibitory effect of Pb stress on the growth of root and shoot lengths of rice. These results were similar to the research results of Chen et al. and Pande et al. [42,43], which indicated that the long-term and short-term release of exogenous NO had different improvement effects on the toxicity of heavy metals in plants.

Moreover, relative to the application of GSNO-CS-NPs, root length and shoot length in the group adding GSNO-CS@S-nZVI/BC were the closest to those in group CK among the treatment groups, which further indicated that the CS@S-nZVI/BC-embedded GSNO achieved a more effective alleviation of Pb stress compared to the treatment group of single CS nanomaterial. Notably, the growth effect of different treatments on rice root length was not as significant as that of shoot length; this could be attributed to Pb being mainly accumulated in the rice root system to protect the aerial part of rice [44].

The weight of rice is shown in Figure 4c. The growth of rice was affected after the addition of Pb, resulting in a significant reduction of 71.2% in fresh weight and 55.6% in dry weight compared to the control group (CK). Furthermore, the results showed that the addition of GSNO significantly increased the biomass of rice compared to the Pb treatment group (*p* < 0.05). It was also evident that, as the concentration of GSNO increased, the biomass of rice also increased. Analogous research results by Liu et al. [45] also indicated that within a certain range, as the concentration of SNP increased, the biomass of plants under heavy metal stress could be enhanced. Compared with the Pb treatment, the addition of CS@S-nZVI/BC significantly alleviated the Pb stress on rice, increasing its fresh weight and dry weight (*p* < 0.05), which might lie in the interaction of its internal components [35]. Furthermore, after embedding GSNO with CS nanoparticles, the increase in dry weight and fresh weight of rice was significantly greater than that of the group that applied a lower dose of GSNO (0.5 g/L) (*p* < 0.05). However, compared with the treatment group only using 1 g/L GSNO, the effect of dry weight and fresh weight by GSNO-CS-NP material treatment became less significant. Studies showed that, as the duration of NO action increased, the plant height and biomass of soybeans under drought stress conditions significantly increased [19]. It was indicated that the slow release of NO could enhance the stress resistance of plants. Additionally, another study had demonstrated that GSNO could alleviate Pb toxicity in soybean by modulating reactive oxygen species (ROS) and metal-related transcript levels [46]. These results demonstrated that both GSNO and the GSNO-encapsulated CS-based nanomaterials effectively enhanced plant stress resistance. However, the increase in dry weight and fresh weight by the GSNO-encapsulated nanomaterials was slightly higher than that achieved by using GSNO alone. The reason might be that CS nanomaterials had played a role in alleviating the effects of Pb stress on dry weight and fresh weight. It was notable that GSNO-CS@S-nZVI/BC and GSNO-CS-NP treatment groups had similar effects on fresh and dry weight of rice plants.

Based on Figure 4b, the root length was similar, but the length of the shoot showed a more significant difference between the GSNO-CS@S-nZVI/BC and GSNO-CS-NP treatment groups. The possible reason was that the composite material fixed a portion of the Pb ions at the root, hindering their transportation to the shoot. However, the growth of the shoot had little impact on the biomass of rice, so the weight change was not significant. Particularly, the change in fresh weight of rice after each group of treatments was more significant than that in dry weight, which might be due to the fact that water uptake of rice was related to the morphology of roots [47]. The addition of GSNO-CS@S-nZVI/BC enhanced the water absorption capacity of rice by promoting the growth of rice roots, an effect mediated by its constituent Fe and the NO released from GSNO [48,49].

#### 2.3.2. Changes in Chlorophyll Levels of Rice in Hydroponic Experiment

Chlorophyll is the basic substance for normal photosynthesis in plants, and the chlorophyll levels of plant leaves can largely reflect the level of photosynthesis in plants. Studies have shown that Pb stress leads to a decrease in chlorophyll b and total carotenoid content in rice, which might be related to the impact on chloroplast function [50]; thus, the chlorophyll levels in plant leaves were also an important basis for reflecting the degree of Pb stress on plants. Figure 4d shows the chlorophyll level of rice in each treatment group. After adding a certain amount of Pb, the chlorophyll levels in the rice leaves showed a significant decrease (*p* < 0.05). When different concentrations of GSNO (0.5 g/L and 1 g/L) were applied, the chlorophyll levels of rice under Pb stress were significantly alleviated, and the alleviating effect was enhanced with the increase in GSNO concentration (*p* < 0.05). Nusrat Jahan et al. [46] reported that GSNO had a beneficial effect on the growth of Pb-stressed soybean seedlings compared to control plants exposed to Pb only, and it increased the chlorophyll levels of Pb-stressed soybean seedlings, which was consistent with the experimental results of the present study. When the CS@S-nZVI/BC composite material was applied to the rice under Pb stress, the chlorophyll levels in the rice were significantly increased (*p* < 0.05), indicating that the composite material could also markedly alleviate the toxic effects of Pb on the photosynthesis of rice. Studies reported that CS maintained the chlorophyll levels of plants by eliminating ROS and through other means [51]. Meanwhile, S-nZVI [52] and BC [53] could also effectively enhance the chlorophyll levels of rice under heavy metal stress. Compared with the treatment groups of GSNO and CS@S-nZVI/BC alone, the GSNO-CS@S-nZVI/BC treatment significantly mitigated the effect of Pb stress on chlorophyll levels in rice. The treatment groups of GSNO were stronger than the CS@S-nZVI/BC group alone. This effect could be primarily attributed to the key role of NO in enhancing chlorophyll levels. The increased GSNO concentration likely extended the interaction time between NO and rice plants through sustained release, thereby promoting greater chlorophyll accumulation [54]. Improving photosynthesis was the main way to increase the yield potential of crops [55]. In correlation with the results of Figure 4c, the highest chlorophyll level was observed in the GSNO-CS@S-nZVI/BC treatment group, which might be due to the fact that nZVI could provide iron to rice, promote the production of photosynthetic pigments in plants, and effectively enhance photosynthesis. Moreover, the application of exogenous NO could increase the reactive iron content and chlorophyll levels of the leaves and stimulate photosynthesis [56].

#### 2.3.3. Distribution of Pb Content in Rice Roots and Shoots in Hydroponic Experiment

The Pb content in the roots and shoots of rice is shown in Figure 4e. In all treatment groups, the Pb concentration in the roots was significantly higher than that in the shoots (*p* < 0.05), indicating that Pb primarily accumulated in the rice root system. This accumulation might alleviate the adverse impacts of Pb on the physiological and biochemical processes of the aboveground parts of rice. Compared to the single Pb treatment group, the addition of composite materials and the release of NO (including the GSNO treatment group, GSNO encapsulated in CS, or the CS@S-nZVI/BC treatment group) markedly decreased the Pb levels in both the root system and shoots of rice (*p* < 0.05). Among them, the GSNO-CS@S-nZVI/BC treatment group showed the greatest reduction effect on the Pb accumulation in rice. Additionally, compared to the GSNO-CS-NP treatment group, the Pb concentration in the solution was significantly lower in the GSNO-CS@S-nZVI/BC treatment group. This could be attributed to the removal effect of composite materials on Pb from the solution, reducing the total amount of Pb entering the rice, thereby lowering its bioavailability. Meanwhile, the sustained release of NO promoted rice growth, and the increase in rice biomass to some extent diluted the Pb levels.

As shown in the NO-free release group, an increase in GSNO content significantly reduced the Pb concentration in the root system. Therefore, subsequent potted experiments might consider adding a moderate amount of GSNO. Additionally, single-factor variance analysis indicated that the sustained release of exogenous NO markedly reduced the accumulation of Pb in hydroponically grown rice (*p* < 0.05). After Pb treatment alone in Figure 4e, the Pb content in the root system significantly increased (*p* < 0.05), indicating that the accumulation of heavy metals inhibits the growth of the root system and further showing that the root system of rice is the most important site for Pb accumulation [57].

In summary, the application of GSNO-CS@S-nZVI/BC resulted in a significant decrease (*p* < 0.05) in Pb accumulation in both roots and shoots compared to the single Pb treatment (Figure 4e). Furthermore, it significantly alleviated the inhibitory effect of Pb stress on the growth of rice roots and shoots (*p* < 0.05) (Figure 4b), as well as mitigated the decrease in chlorophyll content in rice leaves under Pb stress (Figure 4d). This phenomenon could be attributed to the adsorption efficiency of CS@S-nZVI/BC material on Pb, which reduced Pb accumulation in rice roots and its translocation to the aboveground parts, thus stimulating root development. Meanwhile, the sustained release of exogenous NO from GSNO embedded in the CS@S-nZVI/BC material also improved photosynthetic efficiency and increased chlorophyll levels.

### 2.4. NO Sustained Release for Pb Stress Alleviation in Potted Experiment

Based on the beneficial effects of sustained-release NO observed in hydroponic experiments on the growth characteristics, chlorophyll level changes, and Pb distribution in root–shoot partitioning of rice under Pb stress, a potted experiment in soil was conducted to evaluate the practical application value of this system.

#### 2.4.1. Occurrence Form of Pb in Potted Experiment

**Figure 5:**
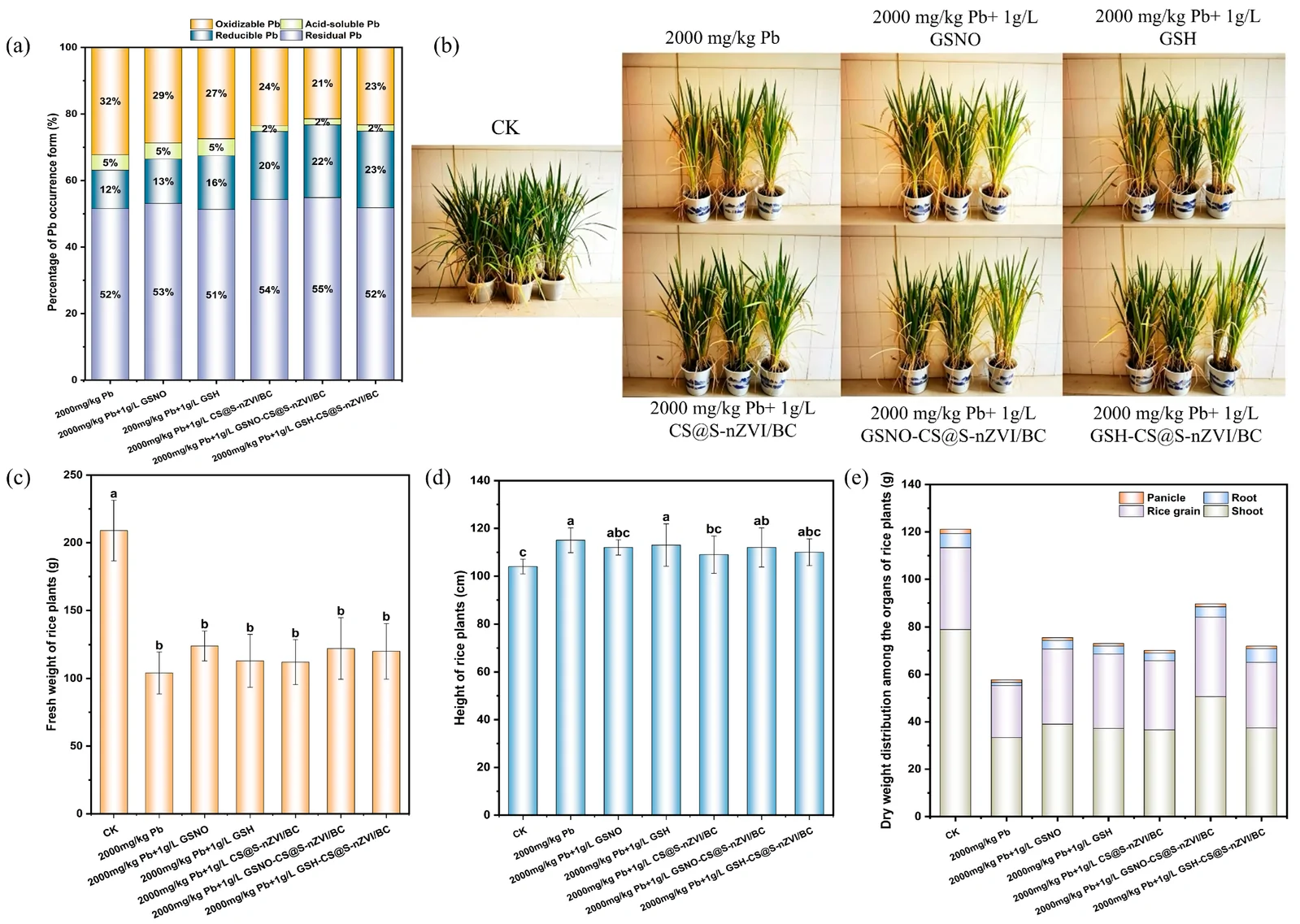
Percentage of Pb occurrence form in potted experiment in the soil (**a**); growth characteristics of rice seedlings under potted conditions (**b**); fresh weight of rice (**c**), height of the rice (**d**), and the dry weight distribution among the organs of rice (**e**) in potted experiment. Different lowercase letters above the bars indicate statistically significant differences (*p* < 0.05). Bars sharing the same letter are not significantly different.

Figure 5a shows the percentage of each speciation of Pb in the soil. The acid-soluble fraction of heavy metals refers to metal ions that exist in exchangeable, adsorbed, or carbonate-bound forms. Owing to their environmental sensitivity and high mobility, these metals are easily bioaccessible and can be directly utilized by organisms.

Compared to the single Pb treatment group, after the application of CS@S-nZVI/BC and GSNO-CS@S-nZVI/BC treatment, the residual Pb percentage increased by 2% and 3%, respectively. Both the addition of CS@S-nZVI/BC and GSNO-CS@S-nZVI/BC reduced the content ratios of oxidizable and acid-soluble Pb, while the proportion of residual Pb, especially reducible Pb, increased significantly. Additionally, the GSH-CS@S-nZVI/BC treatment group also showed a similar effect on Pb speciation distribution to the CS@S-nZVI/BC and GSNO-CS@S-nZVI/BC treatment groups. These results could be attributed to the synergistic effect between BC and S-nZVI, suggesting that the application of CS@S-nZVI/BC further diminished the plant-available fraction of Pb and altered its distribution among various forms in the soil, which was consistent with the findings of Bogusz and Oleszczuk [58].

Additionally, the application of GSNO-CS@S-nZVI/BC was more effective in reducing the proportion of oxidizable Pb and increasing the percentage of residual Pb compared to the application of GSH-CS@S-nZVI/BC. The use of NO increased the available Fe content in the root system, enhanced the availability of iron in the soil, and thus promoted the transformation of heavy metals [59].

#### 2.4.2. Growth Characteristics of Rice in Potted Experiment

As shown in Figure 5b, under the same sunlight irradiation angle, it was observed that the shoot in the single Pb treatment group showed yellowish and shriveled characteristics compared with those of the control group (CK). The plants were relatively thin in the single Pb treatment group, while the shoot of the rest of the groups was mostly bright green. These results indicated that Pb was transported through the root system to the shoot, which impaired the ability of the aboveground parts of rice to produce chlorophyll, resulting in yellowish leaves.

The content graphs of fresh weight and plant height of rice, as well as the dry weight distribution among the organs under Pb stress, are shown in Figure 5c–e, respectively. As shown in Figure 5c, compared to the CK group, it is observed that the fresh weight of rice in the single Pb treatment group is significantly inhibited (*p* < 0.05). However, the results demonstrated that, compared to the single Pb treatment group, the effects of the remaining treatment groups on fresh weight were not significant. It indicated that the addition of the composite material and GSNO did not have a significant impact on the fresh weight of rice under Pb stress. Unlike the hydroponic situation, the moisture in the potted environment was insufficient to meet the growth requirements of rice, which might have led to the phenomenon that the change in fresh weight of the rice was not obvious after adding the composite material and GSNO in the potted experiment.

Furthermore, according to Figure 5d, there are no substantial differences in plant height among the groups, with an average height of approximately 115 cm, indicating that the plant height and overall morphology of rice are not markedly affected by the concealment of Pb pollution. However, although the plant height was almost identical across all treatment groups, there was a significant difference in fresh weight (Figure 5c), which indicated that biomass was a more sensitive indicator than plant height. This result could be attributed to the impairment of photosynthesis in rice under Pb stress, which was characterized by the reduction in chlorophyll content. Such impairment directly inhibited the synthesis of carbohydrates, a key substrate for biomass accumulation [60]. The findings of this study were consistent with those reported by Khan et al. [61] and Ghouri et al. [62]. As shown in Figure 5e, the CK group had the highest total dry weight, at 121.1 g per plant. Compared with the CK group, the total dry weight of each plant after single Pb treatment was only 57.7 g, with an inhibition rate of up to 52.35% (*p* < 0.05); this indicated that Pb could significantly affect the dry weight of rice. After treatment with GSNO and CS@S-nZVI/BC, the total dry weight of rice was relieved to varying degrees by Pb stress compared with the single Pb treatment group, indicating that the application of both groups reduced the toxicity of Pb to rice to varying degrees. Furthermore, the addition of GSNO-CS@S-nZVI/BC had the most significant effect on reducing the dry weight of rice under the influence of Pb (*p* < 0.05) compared to other treatment groups. Therefore, compared to the combination of CS@S-nZVI-BC and GSH, the CS@S-nZVI-BC with GSNO had a greater effect on the total dry weight of rice, suggesting that it was more effective in reducing the toxicity of Pb in rice. Additionally, sustained release NO in the GSNO-CS@S-nZVI/BC treatment group compared to the free-release NO treatment group could be more effective in mitigating the effects of Pb on the stress of the rice in soil.

Among all treatments, GSNO-CS@S-nZVI/BC was the most effective in mitigating the impact of Pb stress on the dry weight of the shoot (Figure 5e). Compared with the single Pb treatment group, the dry weight of the shoot in the single GSNO and GSNO-CS@S-nZVI/BC treatment groups increased by 44.09% and 51.81%, respectively. According to a study by Elsayed Fathi [63], the accumulation of Pb^2+^ within rice led to the generation of large amounts of reactive oxygen species, which subsequently impeded the plant’s absorption of essential elements such as proteins, calcium, zinc, and iron. This deficiency in vital nutrients adversely affected the growth of rice. By using the exogenous NO donor of GSNO, the nutrient input channel could be reopened to continue providing nutrients to the shoot of the plants [64]. Therefore, under single GSNO treatment and GSNO-CS@S-nZVI/BC treatment, the dry weight of the shoot in rice was less affected by Pb stress.

#### 2.4.3. Uptake, Translocation, and Accumulation of Pb in Rice in Potted Experiment

**Figure 6:**
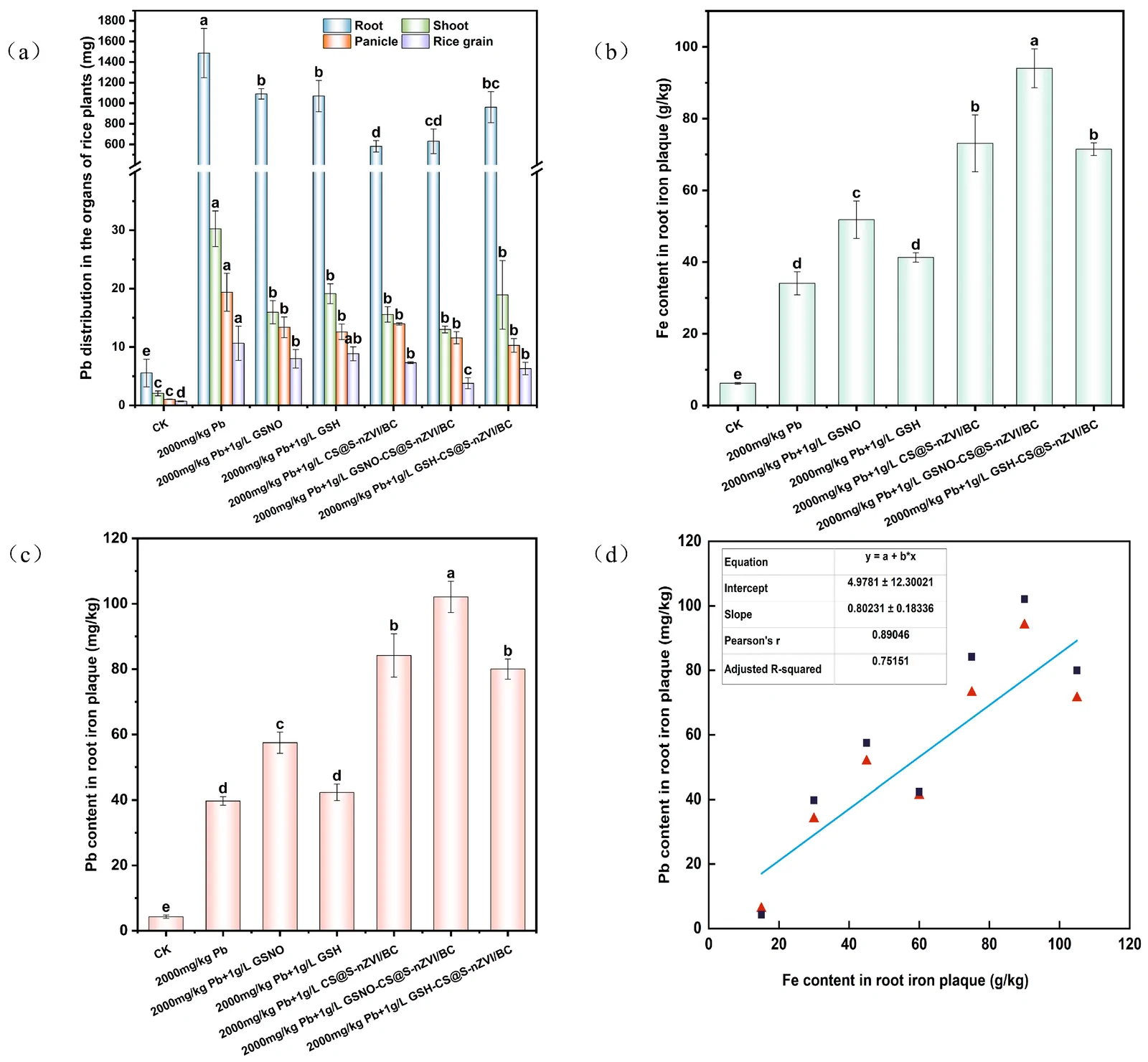
Distribution of Pb in the organs of rice in potted experiment (**a**); the content of Fe (**b**), the content of Pb (**c**), and the correlation between Fe content and Pb content (**d**) in root iron plaque in potted experiment. Different lowercase letters above the bars indicate statistically significant differences (*p* < 0.05). Bars sharing the same letter are not significantly different. Red triangles represent Fe content and blue squares represent Pb content in the root iron plaque.

Figure 6a shows the distribution of Pb in rice plants (roots, shoots, panicles, and rice grains). As seen in Figure 6a, after applying 2000 mg/kg Pb, the Pb content in each part of rice significantly increases (*p* < 0.05), among which the Pb content in the root is the highest and is much higher than the total Pb content of all aboveground parts, indicating that the root system of rice has a strong adsorption capacity for Pb. Figure 6a reveals a wide variation in root Pb content among the treatment groups, ranging from 5.5 to 1486.2 mg/kg. In contrast, the Pb content in the shoot was observed to be lower, ranging from 2.1 to 239.5 mg/kg. The Pb content range was relatively smaller in the panicles and rice grains, with Pb concentrations in the rice panicles ranging from 1.0 to 19.4 mg/kg and in rice grains ranging from 0.7 to 10.6 mg/kg. Compared to the single Pb treatment, the exogenous GSNO treatment could significantly reduce the Pb content in different parts of rice (*p* < 0.05). This phenomenon might be attributed to the fact that exogenous NO promoted the formation of iron plaques on the root surface and combined with Fe^2+^ iron to form nitroso-iron compounds covering the rice roots [65]. These iron plaques immobilized Pb in the roots, thereby blocking Pb absorption by rice roots and reducing its transport to the aboveground parts. In addition, NO was involved in many cellular processes and could act as a signaling molecule, rapidly crossing the cell membrane and triggering various responses to various factors, alleviating various stress effects in plants [66].

Consistent with the effect of GSNO, the application of GSH also mitigated the impact of Pb contamination on various rice organs, indicating its role in restricting Pb distribution. Our results thereby supported the notion that exogenous GSH could mitigate Pb accumulation in plants, as previously demonstrated in Broccoli seedlings [67]. Compared to the GSH-CS@S-nZVI/BC treatment, the GSNO-CS@S-nZVI/BC treatment demonstrated the most effective in decreasing Pb content (*p* < 0.05) across all parts of the rice, suggesting that embedding GSNO within CS@S-nZVI/BC was more effective at reducing Pb content in different parts of the rice compared to embedding GSH.

Notably, compared to the single GSNO treatment, the application of GSNO-CS@S-nZVI/BC treatment significantly reduced Pb in the rice roots (Figure 6a). This might be because the synergistic effect of CS@S-nZVI/BC and NO sustained release was more effective in controlling Pb levels in rice than GSNO alone releasing NO. Studies demonstrated that BC could fix Pb in the root and rhizosphere soil, thereby reducing the toxicity of Pb to the stems of rice [68]. Moreover, our previous studies had shown that CS@S-nZVI/BC had a strong adsorption effect on Pb [35]. In this study, the application of CS@S-nZVI/BC did indeed significantly reduce the content of Pb in rice (*p* < 0.05). Moreover, the research by Mostofa et al. confirmed that exogenous NO could inhibit the uptake and accumulation of heavy metal in rice by forming NO-heavy metal complexes in the nutrient medium [41], further indicating the role of NO in reducing the Pb content in rice.

Bioconcentration factor (BCF) is commonly used to indicate the ability of different plant parts to accumulate heavy metals from the root to the shoot. Table 2 shows the BCF of Pb for different parts of rice in the potted experiment. From Table 2, the shoot exhibits the highest accumulation capacity, followed by the panicle, while the grain has the lowest accumulation capacity. Compared with the single Pb treatment, the treatment of GSNO and GSH both resulted in a significant decline in the BCF of Pb in the shoot, panicle, and rice grain (*p* < 0.05). Moreover, the BCF of the shoot and rice grain was lower after the application of GSNO compared to GSH, indicating that the free release of NO by GSNO was more effective in declining the Pb bioaccumulation in the shoot and rice grain of rice than the use of GSH. The GSNO-CS@S-nZVI/BC treatment group (sustained release of NO) proved more effective than the GSNO treatment group (free release of NO), significantly lowering the BCF in rice shoots, panicles, and grains (*p* < 0.05). The results showed that the sustained release of NO was more effective in reducing the BCF of Pb in the shoots, panicles, and grains of rice. Additionally, compared with the amendment of GSH-CS@S-nZVI/BC, GSNO-CS@S-nZVI/BC treatment was more effective in reducing the bioaccumulation of Pb in the shoot and rice grain of rice, confirming that the synergistic effect of CS@S-nZVI/BC combined with GSNO was more effective in decreasing the BCF of Pb in the shoot and rice grain than its combination with GSH.

In experiments, the translocation factor (TF) is commonly used to indicate the ability of plants to transport heavy metals from the underground parts to the aboveground parts. Table 3 shows the translocation factors for different parts of rice in the soil pot experiment treatment, which was divided into four sections: from soil to root, from root to shoot, from shoot to panicle, and from panicle to rice grain.

Compared with the control group, Pb treatment resulted in a significant increase in the TF from soil to root (*p* < 0.05). However, there were no significant differences observed in the TF from shoot to panicle and from panicle to rice grain (*p* > 0.05), which might be because the majority of Pb was retained in the roots, with only a small portion being translocated to the aboveground parts. It indicated that the root system was the main organ for plants to absorb Pb from the external environment. Zakaria et al. [69] also reported similar findings. Compared with the single Pb treatment group, the inhibition rates of TF from soil to root in the GSNO, GSH, CS@S-nZVI/BC, GSNO-CS@S-nZVI/BC, and GSH-CS@S-nZVI/BC treatment groups were 34.8%, 34.18%, 75.48%, 84.41%, and 74.83%, respectively. It was worth noting that the treatment groups using composite materials all achieved satisfactory results. This might be because each component in the composite material could effectively fix heavy metals and restrict their transfer [70,71,72]. Moreover, the nZVI-BC part could also convert the unstable heavy metals into stable forms, thereby reducing the availability of heavy metals [73]. The reduction in TF from root to shoot ranged between 92.08 and 96.21%, with no significant differences observed among the groups, suggesting that most of the Pb in the rice plants was retained in the roots, and each treatment group was able to effectively reduce TF_shoot/root_. Research has found that the use of NO could reduce the heavy metal concentration in the aboveground parts of rice, thus inhibiting the transport of heavy metals [45]. Additionally, the application of GSH resulted in similar values to the single GSNO treatment, for TF from soil to root and TF from root to shoot in rice, which might be attributed to exogenous glutathione isolating more Pb on the cell walls, thereby reducing Pb translocation through the rice root system [74]. Notably, compared with the addition of GSH-CS@S-nZVI/BC, the application of GSNO-CS@S-nZVI/BC showed better performance in decreasing the TF from soil to root and TF from root to shoot for Pb in rice. Based on the results from the previous studies, it could be speculated that embedding GSNO in the composite material enabled the slow release of NO, which was more effective in reducing TF of Pb in rice compared to GSH.

#### 2.4.4. Formation of Iron Plaque on Rice Roots and Its Impact on Pb Accumulation in Potted Experiment

The amount of Fe generated in the rice root iron plaques is displayed in Figure 6b, and the amount of Pb adsorbed by the iron plaques is shown in Figure 6c. Compared to the single Pb treatment (Figure 6b), the addition of GSNO, GSH, CS@S-nZVI/BC, GSNO-CS@S-nZVI/BC, and GSH-CS@S-nZVI/BC promoted the formation of Fe species in the root iron plaques of rice at different levels. Research has shown that, under low iron content, the application of exogenous NO could effectively enhance the ferric-chelate reductase (FCR) activity, which promoted the absorption of iron by rice from the environment [75]. Furthermore, the addition of CS@S-nZVI/BC also resulted in a notably high Fe content in the rice root, second only to the GSNO-CS@S-nZVI/BC treatment. This could be attributed to the aerenchyma in the rice root system, which constantly supplied oxygen, thereby oxidizing Fe species and leading to the precipitation of iron plaques on the root surface [75]. Therefore, CS@S-nZVI/BC not only provided exogenous iron to the soil environment and raised the pH in soil but also increased radial oxygen loss (ROL) in rice roots, thereby increasing the iron plaque content on the surface of rice roots [76]. Additionally, it could also enhance the formation of iron plaques by releasing S^2−^, SO_4_^2−^, and Fe^3+^ [77,78,79].

Compared to the treatment of GSH, the application of GSNO released exogenous NO to the rice, significantly increasing the amount of Fe generated in the root iron plaques (*p* < 0.05), thereby also enhancing the adsorption of Pb by the iron plaques. Notably, compared with other groups, the GSNO-CS@S-nZVI/BC treatment group had the best repair effect, which was 2.7 times that of the single Pb treatment group and significantly higher than that of the GSNO treatment group (*p* < 0.05). These results suggested that the combination of GSNO and CS@S-nZVI/BC was more effective in promoting the formation of iron plaque on rice roots and the adsorption of Pb by the iron plaques than the combined effect of GSH and CS@S-nZVI-BC or the free release of NO from GSNO alone.

As can be seen from Figure 6d, there is a significant positive correlation (*p* < 0.01) between the amount of Fe generated in the rice root iron plaques and the amount of Pb adsorbed by the iron plaque, indicating that higher Fe content is correlated with greater Pb adsorption, which is similar to the research results of Zhang et al. [80]. Additionally, this further explained the reasons for the differences in Pb content between the aboveground parts and the roots of rice.

## 3. Materials and Methods

### 3.1. Experimental Reagents

The information on chemicals and materials is described in the Supporting Information (Section S1).

### 3.2. Preparation of GSNO

The synthesis of GSNO was mainly based on the study of Hart T. W. [81]. Under ice bath conditions, a certain amount of GSH (1.53 g, 5 mmol, Beijing Solarbio Science & Technology Co., Ltd., Beijing, China) and NaNO_2_ (0.345 g, 5 mmol, Sinopharm Chemical Reagent Co., Ltd., Shanghai, China) were dissolved in ultrapure water (8 mL, LAN-10-H, Beijing Jiayuan Environmental Protection Technology Co., Ltd., Beijing, China) and 2 mol/L hydrochloric acid (HCl, 2.5 mL, Sinopharm Chemical Reagent Co., Ltd., Shanghai, China) to form a red mixed solution. After stirring the mixture for 40 min, 10 mL of acetone was added, and stirring continued for another 10 min, resulting in a light red precipitate. The obtained precipitate was filtered, washed several times with acetone (Sinopharm Chemical Reagent Co., Ltd., Shanghai, China)and ultrapure water, and then freeze-dried for 24 h. The product was stored at low temperature in the dark for use.

### 3.3. Preparation of CS Nanoparticles Containing GSNO (GSNO-CS-NPs)

The synthesis of GSNO-CS-NPs was carried out using the ionotropic gelation method [9]. A certain amount of CS (Sinopharm Chemical Reagent Co., Ltd., Shanghai, China) was dissolved in acetic acid (1%, Sinopharm Chemical Reagent Co., Ltd., Shanghai, China), and 14.29 mmol/L of GSH (Sinopharm Chemical Reagent Co., Ltd., Shanghai, China) was added to the solution. The mixture was magnetically stirred at room temperature for 90 min. Then, 0.6 mg/mL of tripolyphosphate (TPP, Sinopharm Chemical Reagent Co., Ltd., Shanghai, China) was added dropwise to the CS/GSH solution at a volume ratio of 3:1 (CS–TPP). Finally, the mixture was magnetically stirred for at least 45 min to obtain CS nanoparticles containing GSH (GSH-CS-NPs). Then, an equal molar amount of NaNO_2_ was added to the GSH-CS-NPs dispersion liquid. The solution was uniformly mixed and reacted in the dark for 90 min, and the obtained product was named GSNO-CS-NPs.

### 3.4. Preparation of CS@S-nZVI/BC

The preparation of the CS@S-nZVI/BC material was performed using the method described by Xu et al. [82]. The mass ratio of BC and nZVI was set at 0.25, and Na_2_S_2_O_3_ served as the sulfidation reagent with a molar ratio of sodium dithionite (Na_2_S_2_O_4_, Sinopharm Chemical Reagent Co., Ltd., Shanghai, China)/nZVI of 0.14.

### 3.5. Preparation of GSNO-CS@S-nZVI/BC

The preparation of GSNO-CS@S-nZVI/BC was adapted from the ionotropic gelation method used for GSNO-CS-NPs, with CS replaced by the CS@S-nZVI/BC matrix [9,35,80]. CS@S-nZVI/BC was dissolved in acetic acid (1%), and 14.29 mmol/L GSH was added to the solution. Since GSH was the precursor of GSNO, GSH could be nitrosylated to form GSNO. Therefore, GSH group was set up to serve as a control to illustrate the NO release effect in the GSNO group. After magnetic stirring at room temperature for 90 min, the CS@S-nZVI/BC/GSH dispersion solution was supplemented dropwise with 0.6 mg/mL TPP at a volume ratio of 3:1 (CS–TPP) and subjected to magnetic stirring for an additional 45 min to prepare the dispersion liquid containing GSH-loaded composite nanomaterials (GSH-CS@S-nZVI/BC-NPs). Subsequently, an equal molar amount of NaNO_2_ corresponding to the GSH content was added to the GSH-CS@S-nZVI/BC-NPs dispersion liquid, and the mixture was uniformly mixed. Then, the reaction was carried out in the dark for 90 min to obtain GSNO-CS@S-nZVI/BC.

### 3.6. In Vitro Nitric Oxide Release Kinetics

The kinetics of nitric oxide (NO) release from free GSNO, GSNO-CS-NPs, and GSNO-CS@S-nZVI/BC were evaluated under conditions representative of the hydroponic experiments. Freshly prepared suspensions of each material were diluted in distilled water to a final concentration of 1 g L^−1^ (corresponding to the dosage used in hydroponic treatments). Cumulative NO release was monitored over 72 h at room temperature under dark conditions to prevent photodegradation.

At predetermined time intervals (0, 6, 12, 24, 36, 48, and 72 h), aliquots were withdrawn, and the remaining GSNO concentration was quantified by UV–Vis spectrophotometry (UV-2700, Shimadzu, Kyoto, Japan) at 336 nm using a previously established calibration curve (R^2^ = 0.9996). The amount of NO released at each time point was calculated based on the stoichiometric decomposition of GSNO. All measurements were performed in triplicate, and data are expressed as mean ± standard deviation. This approach allowed direct comparison of the release profiles among free and encapsulated formulations.

### 3.7. Determination of Encapsulation Efficiency of GSNO in GSNO-CS@S-nZVI/BC

To determine the encapsulation efficiency (EE%) of GSNO in the GSNO-CS@S-nZVI/BC composite, the method described by Kondak et al. [36] was adopted with minor modifications. After the nitrosation reaction in the dark for 90 min (as described in Section 3.5), the mixture was centrifuged, and 2 mL of the supernatant was collected. The absorbance spectra of the supernatants were recorded using a UV–Vis spectrophotometer in a 1 cm quartz cuvette at room temperature over the wavelength range of 200–800 nm. The concentration of non-encapsulated (free) GSNO in the supernatant was quantified from a calibration curve constructed with standard GSNO solutions in distilled water, where the characteristic absorbance band of GSNO appeared at λ = 336 nm.

The encapsulated form of GSNO into the GSNO-CS@S-nZVI/BC was prepared by the ionotropic gelation method, adding TPP as described in Section 3.5. After the nitrosation reaction in the dark for 90 min, 2 mL of the supernatant was taken and centrifuged. The amount of GSNO loaded into the composite was calculated by subtracting the free GSNO in the supernatant from the total amount of GSNO added. The encapsulation efficiency was calculated using Equation (1) as follows:

(1) $$EE\%=\frac{\mathrm{amount}\;\mathrm{of}\;\mathrm{GSNO}\;\mathrm{in}\;\mathrm{GSNO}-\mathrm{CS}@S-\mathrm{nZVI}/\mathrm{BC}}{\mathrm{total}\;\mathrm{amount}\;\mathrm{of}\;\mathrm{GSNO}\;\mathrm{added}}\times 100\%$$

All measurements were performed in triplicate.

### 3.8. Scanning Electron Microscopy (SEM)

The morphological characteristics of pure GSNO, the CS@S-nZVI/BC carrier, and the GSNO-CS@S-nZVI/BC composite were examined using field-emission scanning electron microscopy (FE-SEM, ZEISS Gemini series, Oberkochen, Germany). Prior to imaging, powder samples were mounted on aluminum stubs with conductive adhesive tape and sputter-coated with a thin layer of gold to enhance conductivity. Images were acquired using both secondary electron (SE) and InLens detectors at an accelerating voltage of 3.0 kV. Working distances were maintained between 4.8 and 7.6 mm. To comprehensively characterize particle morphology, crystal structure, surface features, and agglomeration behavior, multiple magnifications (500×, 2000×, 5000×, 10,000×, and 20,000×) were employed, with at least two to three representative regions imaged for each sample.

### 3.9. Fourier Transform Infrared (FTIR) Spectroscopy

To verify the successful encapsulation of GSNO and elucidate the chemical interactions and functional group variations within the synthesized GSNO-CS@S-nZVI/BC, FTIR spectroscopy was employed. The spectra of the pristine components and the final GSNO-CS@S-nZVI/BC were recorded using an FTIR spectrometer (Nicolet iS50, Thermo Fisher Scientific, Waltham, MA, USA) equipped with a deuterated triglycine sulfate (DTGS) detector and a KBr beam splitter. Samples were prepared via the standard KBr pellet method and scanned over a wavenumber range of 4000 to 400 cm^−1^ at a spectral resolution of 4.0 cm^−1^. For each measurement, 32 scans were accumulated for both the sample and the background to ensure a high signal-to-noise ratio.

### 3.10. Hydrodynamic Particle Size Analysis

To characterize the physicochemical properties of the materials in aqueous environments, the hydrodynamic diameter and particle size distribution were determined via dynamic light scattering (DLS) using a Zetasizer (Nano ZS, Malvern Panalytical, Malvern, UK). Prior to measurement, the solid samples were dispersed in deionized water (1.0 mg/mL) and sonicated for 15 min to ensure homogeneity. The suspensions were transferred into disposable polystyrene cuvettes and analyzed at 25.0 °C. The optical parameters were strictly configured with a dispersant refractive index (RI) of 1.330, a material RI of 1.59, and a material absorption index of 0.010. The results were reported as size distributions by volume to accurately reflect the physical dimensions of the composites.

### 3.11. Zeta Potential Measurement

To further evaluate the surface charge and colloidal stability, which are critical physicochemical properties governing the environmental and biological behavior of the nanomaterials, zeta potential measurements were conducted using electrophoretic light scattering on the aforementioned Zetasizer. The well-dispersed samples were loaded into clear disposable folded capillary cells and analyzed at 25.0 °C. The dispersant (water) parameters were set to a viscosity of 0.8872 cP and a dielectric constant of 78.5. Each measurement comprised six runs, and the electrophoretic mobility was converted to zeta potential using the Smoluchowski approximation. All particle size and zeta potential measurements were performed in triplicate, and the data were presented as mean ± standard deviation.

### 3.12. X-Ray Photoelectron Spectroscopy (XPS)

X-ray photoelectron spectroscopy (XPS) analysis was performed to investigate the surface chemical states of the materials using a Thermo Fisher ESCALAB Xi+ spectrometer (Thermo Fisher Scientific, Waltham, MA, USA) equipped with a monochromatic Al Kα X-ray source (hν = 1486.6 eV). The binding energies were calibrated against the C 1s peak at 284.8 eV. High-resolution spectra of Fe 2p, N 1s, and S 2p were collected and deconvoluted using Avantage software (v5.9921) to identify the chemical species of iron (Fe^0^, Fe^2+^, and Fe^3+^), nitrogen, and sulfur.

### 3.13. N_2_ Adsorption–Desorption Analysis (BET)

The textural properties of the materials, including specific surface area, pore volume, and pore size distribution, were determined by N_2_ adsorption–desorption measurements at 77 K using a Micromeritics ASAP 2460 analyzer (Micromeritics Instrument Corporation, Norcross, GA, USA). Prior to analysis, the samples were degassed under vacuum at an appropriate temperature to remove adsorbed impurities. The specific surface area was calculated by the Brunauer–Emmett–Teller (BET) method, and the pore size distribution was obtained from the adsorption branch using the Barrett–Joyner–Halenda (BJH) model. Total pore volume was determined from the amount of N_2_ adsorbed at a relative pressure (P/P_0_) close to 1.

### 3.14. Hydroponic Experiment with Rice Seedlings

#### 3.14.1. Hydroponic Experimental Design

The rice seeds used in this experiment were plump and similar in size and shape. The seeds were disinfected with 30% hydrogen peroxide (H_2_O_2_, Yantai Jianshuo Chemical Co., Ltd., Yantai, China) for 15 min, and then washed three times with deionized water. Then, the disinfected seeds were wrapped in moist gauze, arranged neatly in Petri dishes, and placed in the artificial climate chamber (temperature 28 °C, humidity 50%) under dark conditions for germination. Deionized water was added as needed during seed cultivation to maintain moisture and ensure successful germination. After 48 h of light-avoidance cultivation, the Petri dish was opened, and the rice seedlings were observed. When the sprout length reached 1–1.5 cm, 672 seedlings (96 wells * 7 groups) with uniform growth were selected, and they were transferred to a 96-well hydroponic plant box. The preparation of Kumura B nutrient solution was based on the research of Xue [83], and 1L Kumura B nutrient solution was added to the hydroponic plant box for a cultivation period of 12 days. Then, the nutrient solution box was removed from the bottom of the hydroponic plant box, and the nutrient solution was added to the 1 L mark line. At the same time, a certain number of materials and lead nitrate (Pb(NO_3_)_2_; Sinopharm Chemical Reagent Co., Ltd., Shanghai, China) were added and stirred evenly for hydroponic experiments. After 5 days, the seedlings were harvested, and various physiological and biochemical parameters were measured. A detailed description of the other processes of the hydroponic experiment is provided in the Supporting Information (Section S2). As shown in Table 4, the experiment is designed with seven treatment groups.

#### 3.14.2. Hydroponic Experimental Sample Processing and Analysis

After 5 days of treatment, three seedlings with similar growth and characteristics were selected from each group. The seedlings were rinsed three times with running tap water (pH 7.2 ± 0.5) and ultrapure water, respectively. The plants were arranged uniformly, and their morphological characteristics were recorded by taking photos. The fresh weights of the roots, shoot, and whole plants were measured, and the plant height, root length, and shoot length were recorded. The plant samples were then placed in an oven at 105 °C for 30 min to deactivate enzymes. Subsequently, the samples were dried at 70 °C to constant weight, weighed, and stored in sealed bags for further analysis. The methods for the measurement of Pb and chlorophyll content, along with the associated data analysis, are presented in the Supporting information (Section S5).

### 3.15. Potted Experiment with Rice Seedlings

#### 3.15.1. Potted Experimental Design

The soil samples for the potted experiment were collected from the surface layer (0–20 cm) of Yuelu District, Changsha City, Hunan Province, China. The sampling site was situated at 112°50′56.5″ E and 28°1′33″ N, at an elevation of 33 m above sea level. Soil treatment and soil property testing methods are presented in the Supporting Information (Texts S3 and S5). The key properties of the tested soil are shown in Table 5. The tested soil exhibited a neutral pH of 6.15, an organic matter content of 17.21 g/kg, and a total nitrogen content of 0.37 g/kg. Among the detected heavy metals, zinc exhibited the highest concentration (73.36 mg/kg), followed by copper (47.85 mg/kg) and lead (15.35 mg/kg), while cadmium was the lowest (0.15 mg/kg). All measured heavy metal concentrations were below the national risk control standards for agricultural land soil pollution (GB 15618-2018) [84].

Based on the results from the hydroponic experiment, the potted experiment included a treatment with 1 g/L GSNO and a GSH treatment group to assess the mitigating effect of NO on Pb stress. Seven treatment groups were designed, and they are shown in Table 6.

A total of 2000 mg/kg Pb (NO_3_)_2_ solution was prepared and added to buckets containing soil, and then the soil was balanced for about 30 days. During the aging period, a sterilized glass rod was inserted into each bucket to facilitate subsequent sampling or marking. To ensure uniform Pb absorption by the soil, the contents of each bucket were homogenized by stirring every 3 days. When conducting the above process, check the soil samples every two days. If necessary, maintain the water level, keeping it about 5 cm above the soil surface.

Seven days prior to transplanting rice, a nutrient solution was prepared to supplement potassium (K_2_CO_3_, Sinopharm Chemical Reagent Co., Ltd., Shanghai, China), phosphorus ((NH_4_)_3_PO_4_, Sinopharm Chemical Reagent Co., Ltd., Shanghai, China), and nitrogen (CH_4_N_2_O, Dongguan Xunye Chemical Reagent Co., Ltd., Dongguan, China) in the soil. The soil was then allowed to equilibrate for 7 days following the addition of nutrients. During this period, the soil was homogenized once daily with a sterilized glass rod to ensure thorough mixing and uniform absorption of the applied nutrients by the soil.

Vigorous and phenotypically uniform rice seedlings were selected and transplanted following the equilibration period. The arrangement was structured as follows: each treatment group consisted of three pots, each pot had three planting holes, and two seedlings were transplanted into each hole.

After transplantation, the materials for each treatment group were added to the nutrient solution at the respective doses and thoroughly mixed, then applied to the rice plants through root irrigation. Specifically, each material was applied at a dose of 1 g per pot by mixing it into 1 L of nutrient solution. Two supplemental applications of the same dose (1 g of material in 1 L of nutrient solution) were conducted during the 3.5-month growth period, approximately on day 35 and day 70 after transplanting. As each pot contained 4 kg of dry soil, the total mass of material applied to each pot was 3 g, corresponding to a final dose of 0.75 g/kg dry soil. Subsequently, the water level was maintained by replenishing water to approximately 5 cm above the soil surface every morning. Fertilizers were regularly applied around the edges of the containers to ensure adequate soil nutrition and prevent seedling scorching.

Transparent plastic sheeting or nylon netting was used to cover the potted plant area to protect against bird predation and severe weather conditions, with daily inspections to ensure the integrity of the covers. Throughout the growth period of the rice, regular observations were conducted on the condition of the plants. After 3.5 months of cultivation, two supplemental applications of the nutrient solution containing materials were conducted. After the rice matured, samples of the entire rice plant and the rhizosphere soil were collected.

#### 3.15.2. Potted Experimental Sample Processing and Analysis

The methods for collecting and processing soil samples and rice samples are described in the Supporting Information (Section S4).

The determination methods for total Pb in soil, Pb content in various organs of rice, as well as the occurrence form of Pb in soil and the contents of Fe and Pb in root surface iron films are described in the Supporting Information (Section S5).

#### 3.15.3. Data Analysis

A detailed description of data analysis is provided in the Supporting Information (Section S6).

## 4. Conclusions

This study developed a GSNO-encapsulated chitosan–biochar-supported sulfidized nanoscale zero-valent iron composite (GSNO-CS@S-nZVI/BC) that integrates sustained NO release with Pb immobilization for remediation of Pb-contaminated rice–soil systems. The composite achieved a high GSNO encapsulation efficiency of 87.92% and exhibited significantly slower and more sustained NO release over 72 h compared with free GSNO and GSNO-CS-NPs.

In hydroponic experiments, GSNO-CS@S-nZVI/BC effectively alleviated Pb toxicity in rice. Compared with Pb treatment alone, it increased rice dry weight and chlorophyll content by 68.5% and 260%, respectively, while reducing Pb concentrations in roots and shoots by 78.2% and 85.8%. In soil pot experiments, the composite significantly decreased Pb accumulation in roots, shoots, panicles, and grains by 57.6%, 57.0%, 40.3%, and 64.3%, respectively. It also reduced the grain bioconcentration factor by 80.7% (to 0.043 ± 0.007) and the soil-to-root translocation factor by 84.41%. Furthermore, GSNO-CS@S-nZVI/BC shifted soil Pb speciation toward less bioavailable forms, decreasing the oxidizable fraction by 11% and increasing the reducible fraction by 10%.

Mechanistic evidence indicated that the remediation effects were achieved through two complementary pathways. First, sustained NO release from the encapsulated GSNO promoted root iron plaque formation, with Fe content in iron plaques increasing by 176.1% and Pb adsorption by iron plaques increasing by 156.9% under GSNO-CS@S-nZVI/BC treatment. A strong positive correlation was observed between Fe content and Pb adsorption in root iron plaques. Second, the superior performance of GSNO-CS@S-nZVI/BC over free GSNO and GSH-loaded controls demonstrated that prolonged NO availability enhanced rice physiological tolerance to Pb stress. Simultaneously, the CS@S-nZVI/BC matrix provided strong Pb adsorption and stabilization capacity through the synergistic effects of chitosan, biochar, and S-nZVI.

Collectively, these results demonstrate that GSNO-CS@S-nZVI/BC can simultaneously alleviate Pb-induced phytotoxicity and reduce Pb transfer from soil to rice grains under controlled conditions. The findings provide direct experimental support for the proposed mechanisms of iron plaque-mediated Pb sequestration and sustained NO-mediated stress alleviation. Further field-scale investigations are needed to assess its long-term effectiveness, environmental safety, and applicability under diverse soil and climatic conditions.

## Figures and Tables

**Table 1:** BET surface area, pore volume, and pore size parameters of CS@S-nZVI/BC and GSNO-CS@S-nZVI/BC.

Thermophysical Properties	CS@S-nZVI/BC	GSNO-CS@S-nZVI/BC
BET surface area (m^2^/g)	299.22	143.31
Total pore volume (cm^3^/g)	0.484	0.383
Average pore size (nm)	6.47	10.69
BJH peak pore diameter (nm) (Adsorption/Desorption)	2.00/3.95	9.25/10.05

**Table 2:** Bioconcentration factors of Pb in different parts of rice.

Groups	BCF _shoot_	BCF _panicle_	BCF _grain_
B	0.547 ± 0.135 a	0.351 ± 0.105 a	0.224 ± 0.030 a
C	0.249 ± 0.047 bcd	0.213 ± 0.061 b	0.139 ± 0.018 cd
D	0.310 ± 0.034 bc	0.204 ± 0.018 b	0.176 ± 0.027 bc
E	0.172 ± 0.012 cd	0.150 ± 0.010 bc	0.114 ± 0.010 bcd
F	0.083 ± 0.008 d	0.074 ± 0.013 c	0.043 ± 0.007 d
G	0.133 ± 0.067 cd	0.071 ± 0.011 c	0.064 ± 0.005 cd

Table legend: B = 2000 mg/kg Pb, C = 2000 mg/kg Pb + 1 g/L GSNO, D = 2000 mg/kg Pb + 1 g/L GSH, E = 2000 mg/kg Pb + 1 g/L CS@S-nZVI/BC, F = 2000 mg/kg Pb + 1 g/L GSNO-CS@S-nZVI/BC, and G = 2000 mg/kg Pb + 1 g/L GSH-CS@S-nZVI/BC. Different lowercase letters within the same column indicate significant differences at *p* < 0.05. Values followed by the same letter(s) are not significantly different.

**Table 3:** Pb translocation factors of the different parts of rice.

Groups	TF_root/soil_	TF_shoot/root_	TF_panicle/shoot_	TF_grain/panicle_
B	26.252 ± 4.668 a	0.388 ± 0.005 b	0.638 ± 0.060 b	0.666 ± 0.099 a
C	17.116 ± 3.467 b	0.015 ± 0.002 b	0.853 ± 0.161 a	0.684 ± 0.111 a
D	17.278 ± 2.230 b	0.018 ± 0.004 b	0.665 ± 0.125 ab	0.868 ± 0.147 a
E	6.438 ± 0.866 c	0.027 ± 0.005 b	0.880 ± 0.082 a	0.758 ± 0.015 a
F	4.094 ± 1.370 cd	0.022 ± 0.004 b	0.895 ± 0.111 a	0.567 ± 0.015 a
G	6.608 ± 1.898 c	0.023 ± 0.014 b	0.626 ± 0.178 b	0.926 ± 0.184 a

Legend: B = 2000 mg/kg Pb, C = 2000 mg/kg Pb + 1 g/L GSNO, D = 2000 mg/kg Pb + 1 g/L GSH, E = 2000 mg/kg Pb + 1 g/L CS@S-nZVI/BC, F = 2000 mg/kg Pb + 1 g/L GSNO-CS@S-nZVI/BC, and G = 2000 mg/kg Pb + 1 g/L GSH-CS@S-nZVI/BC. Different lowercase letters within the same column indicate significant differences at *p* < 0.05. Values followed by the same letter(s) are not significantly different.

**Table 4:** Experimental design of hydroponic experiment.

Treatment Groups	Pb Concentration (mg/L)	Materials	Concentration of Materials (g/L)
CK(A)	0	/	/
B	500	/	/
C		GSNO	0.5
D		GSNO	1
E		CS@S-nZVI/BC	1
F		GSNO-CS-NPs	1
G		GSNO-CS@S-nZVI/BC	1

**Table 5:** Key properties of the tested soil.

pH	Organic Matter g/kg	Total Nitrogen g/kg	Available Phosphorus g/kg	Available Potassium g/kg	Metal Content mg/kg			
					Pb	Zn	Cu	Cd
6.15	17.21	0.37	1.28	158.23	15.35	73.36	47.85	0.15

**Table 6:** Design of potted experiment.

Treatment Groups	Pb Concentration (mg/kg)	Materials	Concentration of Materials (g/L)
CK(A)	0	/	/
B	2000	/	/
C		GSNO	1
D		GSH	1
E		CS@S-nZVI/BC	1
F		GSNO-CS@S-nZVI/BC	1
G		GSH-CS@S-nZVI/BC	1

## Data Availability

The raw data supporting the conclusions of this article will be made available by the authors on request.

## Supplementary Materials

The following supporting information can be downloaded at: https://www.mdpi.com/article/10.3390/molecules31162761/s1, Section S1: Chemicals and materials; Section S2: The process of hydroponic cultivation experiments; Section S3: Soil treatment methods and conditions; Section S4: Soil and rice samples processing; Section S5: The determination methods of soil cultivation experiment; Section S6: Analytical methods; Figure S1: The particle size distribution curves of CS@S-nZVI/BC.
