# Supplementary material for: Alleviating Pb Toxicity in Rice–Soil Systems by GSNO-Encapsulated Chitosan–Biochar/S-nZVI Composites: Sustained Release of NO and the Stabilization of Pb
Source: Molecules. 2026 Aug 8;31(16):2761. doi: 10.3390/molecules31162761 (PMC13515399; doi:10.3390/molecules31162761)
Supplement: Supplementary file 1 [file molecules-31-02761-s001.zip › molecules-4444357-supplementary.pdf]

Section S1：Chemicals and materials

The rice variety used in the potted experiment was Huang Huazhan, with a full growth period of 136 days. In the hydroponic experiment, the rice variety used was Xiangwanxian 13, with a full growth period of 122 days. All seedings were provided by Hunan Golden Nongfeng Seed Industry Co., Ltd. The Cattails biomass used for preparing BC was collected from Yanghu Wetland in Changsha. CHN_3O_6S, NaOH, and CH_4N_2O were supplied by Beijing Solarbio Science & Technology Co., Ltd, Fengchuan Chemical Reagent Technology Co., Ltd., and Guangdong Xunye Chemical Reagent Co., Ltd., respectively. $HClO_4$ and $Na_2S_2O_4$ were acquired from Shanghai Sigma-Aldrich Co., Ltd. $Pb(NO_3)_2$, $(NH_4)PO_4$, K_2CO_3, $NaNO_2$, CH_3COCH_3, $Na_5P_3O_{10}$, $NaBH_4$, Chitosan, $FeSO_4{\bullet}7H_2O$, C_3H_6O, $Na_3C_6H_5O_7{\bullet}2H_2O$, $NaHCO_3$, C_2H_6O, HCl, H_2SO_4, HNO_3, H_3PO_4, CH_3COOH, $Na_2S_2O_4$, and some other reagents were purchased from Sinopharm Chemical Reagent Co., Ltd. All chemical reagents were of analytical grade.

Section S2: The process of hydroponic cultivation experiments

The hydroponic containers for the rice were 96-well plant hydroponic boxes with dimensions of 127 mm in length, 87 mm in width, and 114 mm in height, and a hole diameter of 6.3 mm.

The hydroponic experiment was conducted in the artificial climate chambers on the second floor of the Life Sciences Building at Central South University of Forestry and Technology.

During the experiment, the nutrient solution was replaced every 3 days to ensure adequate nutrition in the hydroponic solution. The pH of the nutrient solution was adjusted using NaOH and HNO_3 solutions to maintain a value between 5.5 and 6.0. The prepared hydroponic plant boxes were placed in a growth chamber to simulate daily photosynthesis. During the day (6:00-18:00), the lights in the growth chamber were turned on, and then set the temperature to 28 ℃ and the humidity to 99%. At night (18:00-6:00), the lights were turned off, the temperature was adjusted to 22°C, and the humidity remained at 99%. The seedlings were continuously cultivated for 20 days, with the nutrient solution being replaced every 3 days.

Section S3：Soil treatment methods and conditions

The soil was prepared by removing root debris, stones, and other impurities, and then air-dried and sieved through a 10-mesh screen to ensure uniformity. Finally, 4 kg of the prepared dry soil was measured using an electronic scale and poured into each uncovered plastic bucket (Top diameter: 26 cm, Bottom diameter: 19 cm, Height: 19 cm).

The soil cultivation experiment was conducted on the third-floor terrace of the Life Sciences Building of Central South University of Forestry and Technology. The site was exposed to natural sunlight, air, and temperature conditions. The annual average precipitation in this area was approximately 1362.6 mm and the annual average temperature was 17.3 ℃. The climate was characterized as a subtropical monsoon humid climate, with concurrent rainfall and heat, and distinct four seasons.

Section S4：Soil and rice samples processing

The soil sample collection mainly involved collecting soil samples from the soil around the root system, air-drying to a certain weight, removing impurities from the soil, and then packaging them through a 20-mesh sieve and placing them in sterilized polyethylene bags for measurement.

The sampling process of rice involved uprooting the rice along with their roots from the soil and collecting the entire plants. After removing large pieces of soil from the roots, the plants were rinsed with tap water and deionized water to remove surface dust and dirt, and then measured their fresh weight and plant height. The dry weight determination began with air drying, followed by 30 min of drying in an oven at 105 ℃, and then the temperature of oven was reduced to 70 ℃ until a constant weight was achieved. After drying, used scissors to separate the roots, shoots, spikes and grains, and measured the dry weight of each part. The rice grains were first hulled with a small huller (JLGJ 4.5, China) to obtain brown rice and grains; then the rice samples were pulverized in different parts with a plant crusher (JFSJ-100-1, China), and labeled and encapsulated into a sterilized polyethylene bags for testing.

Section S5：The determination methods of soil cultivation experiment

Soil pH was determined potentiometrically by mixing air-dried and sieved (< 2 mm) soil with deionized water at a 1:2.5 soil-to-water ratio (w/v). The suspension was shaken for 30 min and allowed to settle for 1 h before measurement. Organic matter content in soil was determined using the Walkley-Black wet chemical oxidation method. The total nitrogen content was analyzed by the Kjeldahl method. Given the acidic soil condition (with a pH value of 6.15), the Bray-1 solution was used to extract available phosphorus, and the determination was carried out by the ascorbic acid molybdenum blue method. Available potassium was extracted with ammonium acetate (NH_4OAc, pH 7.0) and its concentration was quantified using a flame photometer (AP-1200, China). The total Pb, Zn, Cu, Cd content of the soil was determined by the aqua regia-perchloric acid digestion method using a flame atomic absorption spectrophotometer (TAS-999OF, China). The Pb content of rice grains and roots in rice samples was determined by wet digestion (nitric acid-perchloric acid), and the Pb content of shoots, leaves and panicle was determined by dry ashing digestion (GB/T5009-2003) using a Graphite Furnace Atomic Absorption Spectrophotometer (ICE-3500, USA). For the determination of chlorophyll levels in fresh rice samples, an acetone: ethanol (1:1, v/v) extraction and colorimetric method was used, and the measurements were taken using a UV-Visible spectrophotometer (UV-2700, Japan).

The speciation of Pb in soil samples was analyzed using the community bureau of reference (BCR) sequential extraction method based on the Tessier sequential extraction scheme, and determined by a flame atomic absorption spectrophotometer (model TAS-999OF, China). The extractants and corresponding steps were as follows. For the exchangeable and

reducible fractions, extractants were 0.1 mol/L acetic acid and 0.5 mol/L hydroxylamine hydrochloride. Adjusted the pH to 1.5, added a certain amount of extractant, shaked at constant temperature for 16 h, and then centrifuge and filter. For the oxidizable part, first added 30% hydrogen peroxide. After complete digestion through multiple water bath heating at 85 ± 2 ℃, added a certain amount of ammonium acetate, adjusted to pH = 2, shaked for 16 h, and then centrifuged and filtered. For the residual fractions, the extractants were aqua regia and perchloric acid, and digestion was carried out on an electric heating plate above 160 ℃.

Roots surface iron plaque in rice samples was extracted by Dithionite-Citrate-Bicarbonate (DCB) method. The rice root system was washed with deionized water, and the surface moisture was dried before cutting it from the base. It was then placed in a 100 mL beaker and immersed in 30 mL of a mixed solution containing 0.03 mol/L $Na_3C_6H_5O_7 \cdot 2H_2O$ and 0.03 mol/L $NaHCO_3$ for a reaction period of 10 min. Then, 1 g of $Na_2S_2O_4$ was added, and the mixture was homogenized and soaked at room temperature for 1 h. Afterwards, the extract solution was transferred to a 100 mL volumetric flask. The root sections were rinsed three times with deionized water, and the volume was adjusted to 100 mL. The solution was filtered and used to determine the concentrations of Fe and Pb using a graphite furnace atomic absorption spectrophotometer (ICE-3500, USA).

Section S6：Analytical methods

The experimental data were calculated as the average value of three repetitions. Data analysis was performed using Microsoft Excel 2010 and SPSS 22.0. Analysis of variance used the Duncan multiple comparison method in one-way ANOVA to analyze the differences among the treatments ($P < 0.05$); the Pb content in different parts of rice was expressed as mean ± standard deviation (n = 3).

The bioconcentration factors (BCF) for Pb accumulation in different parts of rice were shown in Equation S1 as follows：

$$BCF_X = \frac{C_X}{C_{soil-Pb}} \quad \text{Equation S1}$$

In the formula, BCFx represents the bioconcentration factors of Pb in the rice shoot, panicles, and grains; Cx indicates the concentration of Pb in the rice plant's shoot, panicles, and grains (mg/kg); Csoil-Pb indicates the concentration of Pb in the soil (mg/kg).

The translocation factors (TF) for Pb in different parts of rice were calculated using Equation S2 as follows：

$$TF = \frac{C_i}{C_r} \quad \text{Equation S2}$$

In the formula, TF represents the translocation factor of Pb between different parts of the rice; Ci represents the Pb content in the destination part (mg/kg); and Cr represents the Pb content in the source part (mg/kg).

The experimental data were represented as the average of three repeated experiments. Data analysis was conducted using SPSS 22.0 software. Variance analysis was performed using one-way ANOVA with Duncan's multiple range test to analyze differences among treatments ($P < 0.05$). The Pb content in different parts of rice was expressed as mean ± standard deviation

(n = 3).

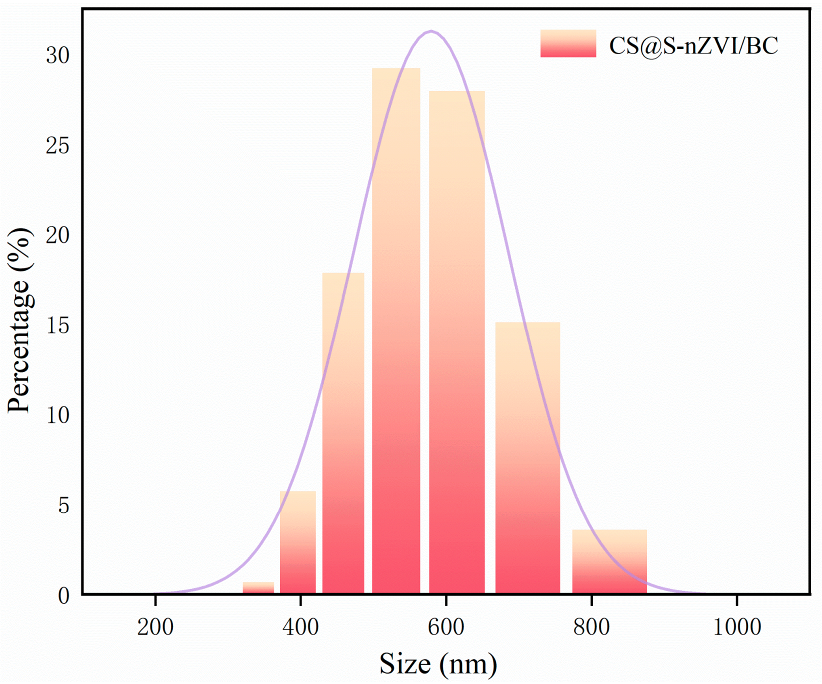

Figure S1. The particle size distribution curves of CS@S-nZVI/BC.
